# Hypoxic‐preconditioned mesenchymal stem cell‐derived small extracellular vesicles promote the recovery of spinal cord injury by affecting the phenotype of astrocytes through the miR‐21/JAK2/STAT3 pathway

**DOI:** 10.1111/cns.14428

**Published:** 2023-08-29

**Authors:** Zhelun Yang, Zeyan Liang, Jian Rao, Haishu Xie, Maochao Zhou, Xiongjie Xu, Yike Lin, Fabin Lin, Chunhua Wang, Chunmei Chen

**Affiliations:** ^1^ Department of Neurosurgery Fujian Medical University Union Hospital Fuzhou Fujian China

**Keywords:** astrocytes, extracellular vesicles, miR‐21, neuroinflammation, spinal cord injury

## Abstract

**Background:**

Secondary injury after spinal cord injury (SCI) is a major obstacle to their neurological recovery. Among them, changes in astrocyte phenotype regulate secondary injury dominated by neuroinflammation. Hypoxia‐preconditioned mesenchymal stem cells (MSCs)‐derived extracellular vesicle (H‐EV) plays a multifaceted role in secondary injury by interacting with cellular components and signaling pathways. They possess anti‐inflammatory properties, regulate oxidative stress, and modulate apoptotic pathways, promoting cell survival and reducing neuronal loss. Given the unique aspects of secondary injury, H‐EV shows promise as a therapeutic approach to mitigate its devastating consequences. Our study aimed to determine whether H‐EV could promote SCI repair by altering the phenotype of astrocytes.

**Methods:**

Rat bone marrow MSCs (BMSCs) and EVs secreted by them were extracted and characterized. After the SCI model was successfully constructed, EV and H‐EV were administered into the tail vein of the rats, respectively, and then their motor function was evaluated by the Basso–Beattie–Bresnahan (BBB) score, Catwalk footprint analysis, and electrophysiological monitoring. The lesion size of the spinal cord was evaluated by hematoxylin–eosin (HE) staining. The key point was to use glial fibrillary acidic protein (GFAP) as a marker of reactive astrocytes to co‐localize with A1‐type marker complement C3 and A2‐type marker S100A10, respectively, to observe phenotypic changes in astrocytes within tissues. The western blot (WB) of the spinal cord was also used to verify the results. We also compared the efficacy differences in apoptosis and inflammatory responses using terminal deoxynucleotidyl transferase dUTP terminal labeling (TUNEL) assay, WB, and enzyme‐linked immunosorbent assay (ELISA). Experiments in vitro were also performed to verify the results. Subsequently, we performed microRNA (miRNA) sequencing analysis of EV and H‐EV and carried out a series of knockdown and overexpression experiments to further validate the mechanism by which miRNA in H‐EV plays a role in promoting astrocyte phenotypic changes, as well as the regulated signaling pathways, using WB both in vivo and in vitro.

**Results:**

Our findings suggest that H‐EV is more effective than EV in the recovery of motor function, anti‐apoptosis, and anti‐inflammatory effects after SCI, both in vivo and in vitro. More importantly, H‐EV promoted the conversion of A1 astrocytes into A2 astrocytes more than EV. Moreover, miR‐21, which was found to be highly expressed in H‐EV by miRNA sequencing results, was also demonstrated to influence changes in astrocyte phenotype through a series of knockdown and overexpression experiments. At the same time, we also found that H‐EV might affect astrocyte phenotypic alterations by delivering miR‐21 targeting the JAK2/STAT3 signaling pathway.

**Conclusion:**

H‐EV exerts neuroprotective effects by delivering miR‐21 to promote astrocyte transformation from the A1 phenotype to the A2 phenotype, providing new targets and ideas for the treatment of SCI.

## Graphical abstract

Compared with EV, H‐EV can inhibit the expression of A1‐type astrocytes and promote the expression of A2‐type astrocytes to reduce neuroinflammatory response, thus promoting SCI repair. Meanwhile, H‐EV may promote the expression of JAK2 and STAT3 by delivering miR‐21, thus promoting the conversion of astrocytes from type A1 to type A2.

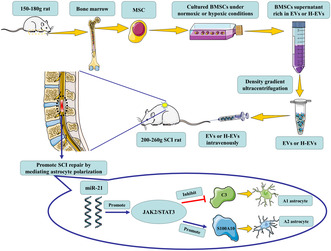

## 1 INTRODUCTION

Spinal cord injury (SCI) is a major problem in the medical community, with high morbidity and mortality, which brings devastating disasters to the physical health of patients and the social economy.^1^ According to incomplete statistics, the mortality rate of hospitalized acute SCI in the world is between 4.4% and 16.7%, and the incidence rate has increased from 16% to 30.5%.^2^ In pathophysiology, SCI is caused by primary injury leading to neuronal and glial cell injury, which then triggers secondary injury dominated by neuroinflammation, resulting in progressive cell death and lesion aggravation.^3^ At present, the clinical treatment of SCI mainly includes early surgical decompression and glucocorticoid pulse therapy.^4^, ^5^, ^6^ However, it is difficult for these treatments to effectively inhibit the secondary injury caused by neuroinflammation and thus promote the recovery of neurological function.^7^ Within the intricate landscape of secondary injury, H‐EV exerts their effects through a multifaceted interplay with various cellular components and signaling pathways. These vesicles have been shown to possess potent anti‐inflammatory properties, dampening excessive immune responses that contribute to secondary injury progression.^8^ Additionally, H‐EV can modulate oxidative stress by delivering antioxidant molecules and enhancing endogenous cellular defense mechanisms.^9^ Furthermore, H‐EV has demonstrated the ability to modulate apoptotic pathways, promoting cell survival and reducing neuronal loss.^10^ Collectively, the unique pathophysiological aspects of secondary injury provide a fertile ground for exploring the therapeutic potential of H‐EV, paving the way for innovative treatment strategies aimed at mitigating the devastating consequences of secondary injury.

Astrocytes are the most abundant cells in the central nervous system and have many functions, such as maintaining the blood–brain barrier and environmental homeostasis, transmitting immune signals, and synaptic signals.^11^, ^12^ In recent years, it has been shown that astrocytes and macrophages are similar, and there are two phenotypes, A1 and A2.^13^, ^14^ During the inflammatory response, lipopolysaccharide (LPS) induces microglia to release cytokines (IL‐1α, TNF‐α, and C1q), which activates astrocytes to transform into a deleterious A1 phenotype, releasing neurotoxic factors such as specific complement components C3 and inflammatory cytokines to induce neuronal and other glial cell death.^15^ Whereas astrocytes are induced to the A2 phenotype under hypoxic–ischemic conditions, they express the specific protein S100A10 and secrete anti‐inflammatory cytokines and neurotrophic factors, thus exerting neuroprotective functions.^16^ SCI can activate astrocytes, and the two types of reactive A1 and A2 astrocytes undergo dynamic temporal and spatial changes during the pathological process.^17^

In recent years, cell therapy has become a popular treatment for neurodegenerative diseases, of which mesenchymal stem cells (MSCs) are no exception and have been widely studied to confirm good efficacy for SCI.^18^, ^19^ Even in clinical trials, some investigators have found good prospects for MSCs in the treatment of SCI.^20^ Despite the remarkable efficacy of cell therapy, there are still some inevitable problems. The risks of transplantation of these cells include poor survival, strong immune rejection, and tumorigenicity, resulting in limited clinical use.^21^, ^22^ Fortunately, recent studies have shown that a large part of the effect of MSCs is due to the role of extracellular vesicle (EV) secreted by them.^23^, ^24^

EVs are nanoscale phospholipid bilayer vesicles secreted by cells containing various proteins and RNAs involved in trafficking communication between cells.^25^, ^26^ More importantly, the efficacy of this cell‐free therapy is similar to that of cell therapy, and it can also overcome the risk of low survival, tumorigenicity, and strong immune rejection of cell transplantation.^27^, ^28^ In recent years, this cell‐free therapy has played a huge role in various fields such as inflammation, tumor, and materials, and is expected to be a new star.^29^, ^30^, ^31^ It has been shown that hypoxia‐preconditioned MSCs are more adapted to the microenvironment after injury in vivo and contribute more to the enhancement of efficacy than MSCs under normoxic conditions.^32^ Moreover, MSCs under hypoxic conditions can mimic the injury state in vivo, and the transplantation of cells in this state in vivo can enhance their biological activity and enhance myocardial repair.^33^ Similarly, it has also been shown that treatment with hypoxia‐preconditioned MSC‐derived extracellular vesicle (H‐EV) can better inhibit neuronal apoptosis and promote angiogenesis than MSC‐derived extracellular vesicles under normoxic conditions (EV).^8^, ^10^, ^34^ At present, a large majority of investigators believe that EV play an important role by delivering miRNA, but the mechanism of their action in a variety of diseases is still unclear.^35^, ^36^, ^37^ Although it has been shown that MSC‐EV can treat SCI by inhibiting neurotoxic astrocytes (A1 type).^38^, ^39^ It has also been shown that up‐regulation of miR‐181c in EV can inhibit the NF‐κB signaling pathway by inhibiting the target gene PTEN, reduce the expression of pro‐inflammatory cytokines (TNF‐α and IL‐1β) secreted by microglia, and alleviate SCI by inhibiting apoptosis and inflammatory response.^40^ However, it is still unclear whether H‐EV could exert better efficacy than EV, especially its effect on the phenotypic changes of astrocytes. If so, whether it can affect the phenotype of astrocytes through the delivery of specific miRNA to promote SCI repair.

Therefore, we attempted to conduct a comprehensive comparative evaluation of the efficacy of EV and H‐EV in SCI, particularly the effect on the phenotypic alteration of reactive astrocytes. By sequencing, we found that miR‐21 was highly up‐regulated in H‐EV, and it has also been shown that the JAK2/STAT3 pathway plays an important role in astrocyte phenotypic changes.^41^ To further investigate whether miR‐21 plays a role in the alteration of astrocyte phenotype, we examined whether miR‐21 promotes SCI repair by modulating astrocyte transformation from A1 to A2 via the JAK2/STAT3 pathway. Our study provides new ideas for the treatment of SCI and provides more selection targets and directions for future research.

## 2 METHODS

### 2.1 Isolation, culture, and identification of BMSCs

BMSCs were isolated from 150 to 180 g Sprague–Dawley (SD) rat bone marrow as described previously.^42^ Briefly, rat femurs and tibias were dissected and bone marrow was flushed with DMEM high glucose medium containing 10% fetal bovine serum (FBS) and 1% penicillin–streptomycin solution (Cat No: SV30010; HyClone). Cells were centrifuged at 300×*g* for 5 min, and the pellet was resuspended in DMEM high glucose medium containing 10% FBS and 1% penicillin/streptomycin solution (Cat No: SV30010; HyClone). Cells were seeded in 75 cm^2^ flasks (Corning) and subsequently cultured at 37°C in a cell incubator with 5% CO_2_ and 21% O_2_. After 24 h, the medium and non‐adherent cells were discarded by changing the medium. Adherent cells were incubated, and the medium was changed every 2 days and passaged when cells reached approximately 90% confluence. When cells were passaged to passage 4, surface proteins Armenian hamster anti‐CD29‐PE (1:200, 12‐0291‐82; eBioscience), mouse anti‐CD90‐APC (1:200, 17‐0900‐82; eBioscience), rat anti‐CD34‐FITC (1:200, 11‐0341‐82; eBioscience), and mouse anti‐CD45‐V450 (1:200, 561,587; BD Biosciences) expression levels identify their purity. Meanwhile, BMSCs were fully identified by differentiation induction using osteogenic (RAXMX‐90021; Cyagen), chondrogenic (RAXMX‐90041; Cyagen), and adipogenic kit (RAXMX‐90031; Cyagen) according to the manufacturer's instructions.

### 2.2 Isolation and identification of EV and H‐EV

When BMSCs reached 80% confluence, the medium was exchanged with a complete medium prepared with 10% exosome‐free FBS (C3801005; ViVaCell), and cultured in normoxic (37°C, 5%CO_2_, and 21%O_2_) or hypoxic (37°C, 5%CO_2_, and 1%O_2_) cell incubators (H35; DWS). The conditioned medium was collected after 48 h, and to remove cell debris, the collected conditioned medium was centrifuged at 300 × g, 4°C for 10 min, then 2000×*g*, 4°C for 10 min, and finally 10,000×*g*, 4°C for 30 min.^43^ After centrifugation, to further purify EV and H‐EV, the supernatant was slowly added to an ultracentrifuge tube containing 4 mL 30% sucrose cushion (Beckman, US) in an ultracentrifuge (Optima XE‐100) at 110,000×*g* for 70 min at 4°C. The upper medium layer was then discarded, and the sucrose layer was retained, diluted in phosphate‐buffered saline (PBS), mixed, and centrifuged again at 110,000×*g* at 4°C for 70 min. Finally, the supernatant was discarded after centrifugation, and the EV and H‐EV pellets were resuspended with 1 mL PBS per centrifuge tube to obtain the EV and H‐EV suspensions. EV and H‐EV were either stored at −80°C or used immediately for downstream experiments.

We observed the morphology of extracellular vesicles obtained under normoxic and hypoxic conditions by transmission electron microscopy (TEM) (HT‐7700; Hitachi). The distribution of vesicle diameters from EV and H‐EV was analyzed using a nanoparticle tracking analysis (NTA) (N30E; NanoFCM). Vesicular protein concentrations of EV and H‐EV were determined using a bicinchoninic acid (BCA) protein assay kit (P0012S; Beyotime), and absorbance was read with a microplate reader (Multiskan MK3; Thermo Fisher Scientific). Specific extracellular vesicle surface markers, such as rabbit anti‐CD9 (1:1000, SAB4503606; Sigma), rabbit anti‐CD63 (1:1000, PA5‐92370; Invitrogen), and mouse anti‐TSG101 (1:1000, MA1‐23296; Invitrogen), were determined by western blotting.^44^

### 2.3 Modeling and grouping of SCI in rats

The animal study protocol was approved by the Animal Committee of Fujian Medical University Union Hospital, and the Ethical Review Form for Animal Experiments was numbered FJMU IACUC 2021‐0501. SD rats were purchased from BeiGene (Beijing) Co., Ltd. (Permit No.: SCXK (Jing) 2019‐0010). All rats were housed in cages with a 12‐h light/dark cycle, ambient humidity of 55–60%, a temperature of 22–24°C, free access to water and food, and were specifically cared for by nursing staff who were completely blind to the experiment. SCI models were developed in adult male SD rats (6–8 weeks old, 200–260 g) as described previously.^45^ After the animals were anesthetized with isoflurane mixed with oxygen (induction concentration 3–4%, maintenance concentration 1.5–2%), the spinal cord was exposed at T10 using a laminectomy and hit with a rod (strike head diameter of 3 mm) at a speed of 0.5 m/s, a depth of 1 mm, and a duration of 1 s using a spinal cord impactor (68099; RWD). Immediately after the strike, the muscle was sutured, the skin closed, and 2 mL of normal saline and 0.1 mL of penicillin sodium were injected subcutaneously for three consecutive days. The bladders of the animals were manually voided twice daily until bladder function recovered. The criteria for successful modeling of SCI were hemorrhage and edema of spinal cord tissue, intact purple and tense and distended dura mater, delayed paralysis of retraction‐like flutter of lower limbs, and spasmodic swing of tail after impact. Inclusion criteria: the rats showed flaccid paralysis of the lower limbs and the BBB score was 0 after awakening from anesthesia.

The rats were randomly divided into four groups (*n* = 30/group). One group was the sham group which underwent laminectomy alone without any treatment. One group was the SCI control group and 500 μL PBS was injected into the tail vein as control after SCI. The other two groups were EV and H‐EV treatment groups, and the equal volume (500 μL) of EV and H‐EV (100 μg of total extracellular vesicle protein dissolved in 500 μL PBS) were injected into the tail vein after the SCI model was established. Dosing time was the day after modeling with SCI, and after the rats recovered from anesthesia, they were randomly divided into groups according to the inclusion criteria and administered for three consecutive days.

The rats were randomly divided into four groups (*n* = 5/group). One group was the H‐EV treatment group overexpressing miR‐21 (miR‐21 up agomir). One group was the H‐EV treatment group with negative control of miR‐21 up agomir (agomir NC). One group was the H‐EV treatment group that knocked down miR‐21 (miR‐21 down antagomir). One group was the H‐EV treatment group with negative control of miR‐21 down antagomir (antagomir NC). 10 μL of miR‐21 up agomir, agomir NC, miR‐21 down antagomir, or antagomir NC at a concentration of 20 μM (GenePharma) and 10 μL GP‐transfect‐Mate transfection reagent (G04009; GenePharma) were used to transfuse miR‐21 up agomir, agomir NC, miR‐21 down antagomir, or antagomir NC at a concentration of 20 μm at room temperature for 20 min. After 20 min co‐incubation at room temperature, nucleic acids, liposomes, and 500 μL H‐EV were finally injected into the tail vein after SCI. The administration plan was the same as above.

### 2.4 EV uptake by CTX‐TNA2 astrocytes

BMSCs were fluorescently labeled according to the manufacturer's instructions. Briefly, BMSCs in the logarithmic growth phase were taken and after washing with PBS, the cell density was adjusted to 1 × 10^6^ cells/mL, and CFSE (65‐0850‐84; Thermo Scientific) was added to a final concentration of 5 μM and labeled at 37°C for 10 min. After extensive washing with PBS, the reaction was terminated by adding DMEM complete medium and incubated at 37°C for 10 min. The cells were then reinoculated into culture flasks, and the supernatant was collected to obtain CFSE‐labeled EVs. Then, these CFSE‐labeled EVs were co‐cultured with CTX‐TNA2 astrocytes (CRL‐2006; ATCC) for 24 h before cells were washed with PBS and fixed in 4% paraformaldehyde. The uptake of CFSE‐labeled EVs by CTX‐TNA2 astrocytes was then observed by an inverted fluorescence microscope (DMI8; Leica).

### 2.5 Grouping of CTX‐TNA2 astrocytes in vitro

While primary cells are derived directly from animal or human tissues and closely resemble the physiological characteristics of the tissue of interest, there are certain advantages to using established CTX‐TNA2 cell lines. First, cell lines are easily accessible from commercial sources, ensuring a consistent supply and reproducibility. Second, the immortalized nature of CTX‐TNA2 allows for long‐term studies and repeated experiments without the need for constantly obtaining new primary cells. Third, cell lines are more homogeneous compared to primary cells, reducing cellular heterogeneity and facilitating focused investigations. Lastly, established CTX‐TNA2 cell lines have well‐documented characteristics, enabling us to design experiments based on prior knowledge and published data. Overall, these advantages support the choice of CTX‐TNA2 cell line and contribute to the experimental efficiency and reliability.

To verify the comparison of the efficacy of EV and H‐EV on astrocyte activation, CTX‐TNA2 astrocytes were divided into four groups (*n* = 3/group). One group was the blank control group (Control), in which the cells were inoculated into six‐well plates, and fresh medium was replaced only at the same time as the other groups without any treatment. One group was the astrocyte conditional medium (ACM) control group (ACM + PBS). C1q (400 ng/mL, MBS147305; MyBioSource), TNF‐α (30 ng/mL, 400‐14; Peprotech), and IL‐1α (3 ng/mL, 400‐01A; Peprotech) were added to DMEM to prepare ACM.^46^ When the density of cells inoculated into six‐well plates grew to 70%, fresh medium was replaced and ACM was added for stimulation at the same time. After 24 h, the cells were washed with PBS. Then, the medium was replaced with fresh medium containing an equal amount of PBS as the control group. The other two groups were the EV (ACM + EV) and H‐EV (ACM + H‐EV) treatment groups. After stimulation with ACM as described above, the medium was replaced with fresh medium containing EV and H‐EV at a final concentration of 20 μg/mL. After 24 h of treatment, all cells and cell supernatants were harvested for subsequent experiments.

To verify the comparison of the efficacy of EV and H‐EV on astrocyte apoptosis, CTX‐TNA2 astrocytes were divided into four groups (*n* = 3/group). One group was the blank control group (Control), in which cells were inoculated into six‐well plates, and fresh medium was replaced only at the same time as the other groups without any treatment. One group was the ultraviolet ray (UVR) control group (UVR + PBS), and when the density of cells inoculated into six‐well plates grew to 70%, fresh medium was replaced and placed on the ultra‐clean table to stimulate with UVR irradiation.^47^ After 2 h, the cells were washed with PBS and replaced with a fresh medium containing an equal amount of PBS as the control group. The other two groups were EV (UVR + EV) and H‐EV (UVR + H‐EV) treatment groups. After UVR stimulation as described above, the medium was replaced with fresh medium containing EV and H‐EV at a final concentration of 20 μg/mL. After 24 h of treatment, all cells and cell supernatants were harvested for subsequent experiments.

To verify whether H‐EV affected the phenotypic changes of CTX‐TNA2 astrocytes by delivering miR‐21, CTX‐TNA2 astrocytes were also divided into four groups (*n* = 3/group). One group was the H‐EV treatment group overexpressing miR‐21 (miR‐21‐H‐EV). When the density of cells inoculated into six‐well plates grew to 70%, fresh medium was replaced and ACM was added for stimulation. After 24 h, the cells were washed with PBS. 5 μL of miR‐21 mimic at a concentration of 20 μM (GenePharma) and 5 μL Hieff Trans liposome nucleic acid transfection reagent (40802ES02; Yeasen) were used to transfuse miR‐21 mimics at a concentration of 20 μm at room temperature for 20 min. After 20 min co‐incubation at room temperature, nucleic acids and liposomes were finally co‐incubated with 100 μL fresh medium of H‐EV at a concentration of 0.2 μg/μL for 24 h with cells. One group was the H‐EV treatment group with negative control of miR‐21 mimics (NC‐H‐EV). After ACM stimulation as described above, 5 μL negative control of miR‐21 mimics at a concentration of 20 μM and 5 μL Hieff Trans liposome nucleic acid transfection reagent were co‐incubated at room temperature for 20 min. Finally, nucleic acids and liposomes were incubated with 100 μL of H‐EV fresh medium at a concentration of 0.2 μg/μL for 24 h with cells. One group was the H‐EV treatment group that knocked down miR‐21 (miR‐21‐IN‐H‐EV). After ACM stimulation as described above, 5 μL of miR‐21 inhibitor at a concentration of 20 μM and 5 μL Hieff Trans liposome nucleic acid transfection reagent were co‐incubated at room temperature for 20 min. Finally, nucleic acids and liposomes were incubated with 100 μL of H‐EV fresh medium at a concentration of 0.2 μg/μL for 24 h with cells. One group was the H‐EV treatment group with negative control of miR‐21 inhibitor (IN‐NC‐H‐EV). After ACM stimulation as described above, 5 μL negative control of miR‐21 inhibitor at a concentration of 20 μM and 5 μL Hieff Trans liposome nucleic acid transfection reagent were co‐incubated at room temperature for 20 min. Finally, nucleic acids and liposomes were incubated with 100 μL of H‐EV fresh medium at a concentration of 0.2 μg/μL for 24 h with cells. After 24 h of treatment, all cells and cell supernatants were harvested for subsequent experiments.

To further validate the targeted regulation of JAK2/STAT3 by miR‐21, CTX‐TNA2 astrocytes were divided into four groups (*n* = 3/group). One group was the blank control group (Control), in which cells were treated in the same way as above. One group was the ACM control group (ACM + PBS), in which cells were treated in the same way as above. One group was the H‐EV treatment group overexpressing miR‐21 (ACM + miR‐21‐H‐EV), in which cells were treated in the same way as above. The last group was the AG490 group (ACM + miR‐21‐H‐EV + AG490). After ACM stimulation as described above, AG490 (HY‐12000; MedChemExpress), an inhibitor of JAK2/STAT3 at a concentration of 10 μM, was added 1 h before treatment with overexpressing miR‐21 H‐EV. After 24 h of treatment, all cells were harvested for subsequent experiments.

### 2.6 Behavioral assessments (BBB score, footprint analysis, electrophysiology)

The BBB score was used to evaluate the recovery of motor function at 1, 3, 7, 14, 21, and 28 days after injury. Scores range from 0 (complete paraplegia) to 21 (normal function).^48^

Footprint analysis was performed on SCI using Catwalk XT (Stones, China), where animals were first trained to walk uninterruptedly on a catwalk track.^49^ On day 28 after SCI, four trials without significant interruption were obtained for each animal as valid runs. Individual footprints were manually determined using runway walk software. Then three gait parameters calculated automatically by the software were selected for analysis. The base of support (BOS) was measured as the width of the area between the left and right hind paws. The stride length was calculated by averaging the two hind paw values. When walking on an uninjured animal, the footprints of the hind paws tend to overlap with those of the fore paws. However, injured animals frequently lose this ability to coordinate between the fore and hind paws. Therefore, relative positions of the fore and hind paws were obtained by directly measuring the distance between the central pads of the ipsilateral fore and hind paw during each step cycle. Regularity index (RI) was used for an objective analysis of gait coordination and calculated from the number of normal step sequence patterns multiplied by four and divided by the total amount of paw placement.

To evaluate the functional recovery after SCI more objectively, the recovery of hind limb muscles was observed using an electrophysiological monitor (Medtronic).^50^ Motor‐evoked potentials (MEP) of SCI rats were analyzed using electromyography (EMG) for 28 days. First, a stimulation electrode was placed subcutaneously in the midline of the skull after anesthesia. Recording electrodes were then placed in the flexor tendon of the biceps femoris. Reference electrodes were inserted into the tendon at the distal end of the muscle of the hind limb. Finally, a ground electrode was placed under the skin of the back. A single square‐wave stimulus (75 μs duration, 50 V intensity, 200 Hz filtering) was applied. The area under curve (AUC) and peak amplitude were used to detect nerve conduction function in the hind limbs of rats. For all of the above behavioral assessments, each rat was evaluated by two independent examiners who were blinded to the treatment regimen.

### 2.7 Histological analysis

On day 28 after SCI, the rats were perfused with 0.9% saline followed by 10% formaldehyde. Spinal segments around the lesion center were removed and fixed overnight in 10% formaldehyde. After stepwise dehydration with different concentrations of ethanol solution, the samples were transparent and wax‐soaked. The samples were embedded in paraffin and cut into 3 μm sections for subsequent experiments. Staining was performed using the hematoxylin and eosin (H&E) staining kit (G1120; Solarbio) according to the manufacturer's instructions. Lesion site size in H&E staining images was analyzed using ImageJ software (Media Cybernetics).

### 2.8 Immunofluorescence (IF)

At 3, 7, and 14 days after SCI, the rats were perfused with 0.9% saline followed by 10% formaldehyde. Spinal segments around the lesion center were removed and fixed overnight in 10% formaldehyde. After stepwise dehydration with different concentrations of ethanol solution, the samples were transparent and wax‐soaked. The samples were embedded in paraffin and cut into 3 μm sections for subsequent experiments. For immunofluorescence staining of tissues, sections were blocked with blocking buffer (1 × PBS/5% BSA/0.5% Triton™X‐100) for 1 h at room temperature. Primary antibodies were diluted in dilution buffer (1 × PBS/1% BSA/0.5% Triton™X‐100). The following primary antibodies were used: mouse anti‐GFAP (1:100, 14‐9892‐82; Invitrogen), rabbit anti‐S100A10 (1:500, PA5‐95505; Invitrogen), and goat anti‐C3 (1:500, PA1‐29715; Invitrogen). Tissue sections were incubated with primary antibodies overnight at 4°C, washed three times with PBS, and incubated with donkey anti‐goat AF647 (1:200, ab150135; Abcam) fluorescent secondary antibodies for 1 h at room temperature and in the dark. The tissue sections were then incubated with goat anti‐mouse AF488 (1:200, A32723; Invitrogen) and goat anti‐rabbit AF568 (1:200, A11010; Invitrogen) fluorescent secondary antibodies for 1 h at room temperature and in the dark after washing three times with PBS. After the final three washes with PBS, the slides were sealed with a DAPI‐containing anti‐fluorescence quenching agent (P0131; Beyotime). The slides were observed and photographed using OlyVIA VS200.

### 2.9 Terminal deoxynucleotidyl transferase dUTP terminal labeling (TUNEL) staining method

On day 7 after SCI, the rats were perfused with 0.9% saline followed by 10% formaldehyde. Spinal segments around the lesion center were removed and fixed overnight in 10% formaldehyde. After stepwise dehydration with different concentrations of ethanol solution, the samples were transparent and wax‐soaked. The samples were embedded in paraffin and cut into 3 μm sections for subsequent experiments. After the paraffin sections were deparaffinized, 20 μg/mL proteinase K without DNase (ST532; Beyotime) was added drop by drop, incubated at room temperature for 30 min, and then washed three times with PBS. The one‐step TUNEL apoptosis detection kit (green fluorescence) was then used for detection (C1086; Beyotime). TUNEL assay solution was dropped and incubated at 37°C in the dark for 60 min and then washed three times with PBS. Finally, the slides were sealed with an anti‐fluorescence quenching mounting solution containing DAPI and observed under an inverted fluorescence microscope (DMI8; Leica). Apoptotic cells and total cells were counted in three randomly selected fields, and the proportion of TUNEL‐positive (apoptotic) cells was calculated. Then, the average value was calculated, and the statistical analysis was performed according to the number of cells in each group.

### 2.10 Flow cytometry

The cell suspension was centrifuged at 300×*g* for 5 min, and CTX‐TNA2 astrocytes were collected, resuspended in PBS, and centrifuged again. This step was repeated twice to wash the cells. The cells were then ruptured and fixed using the Fix/perm kit (GAS004; Invitrogen) according to the manufacturer's protocol, and then incubated with primary antibodies rabbit anti‐S100A10 (1:100, PA5‐95505; Invitrogen) and goat anti‐C3 (1:100, PA1‐29715; Invitrogen) at 4°C for 30 min. After washing three times with PBS, cells were incubated at 4°C for 30 min with the secondary antibodies donkey anti‐rabbit PE (1:200, 12‐4739‐81; Invitrogen) and donkey anti‐goat AF647 (1:200, Abcam, ab150135). After washing twice with PBS, all samples were then analyzed with a flow cytometer (FACS Calibur; BD Biotechnology). At least 2 × 10^4^ cells were analyzed in each sample.

The apoptosis state of CTX‐TNA2 astrocytes was detected using an Annexin V‐FITC apoptosis detection kit (C1062L; Beyotime). The cell suspension was resuspended in PBS and centrifuged, and this step was repeated twice to wash the cells, followed by centrifugation at 300×*g* for 5 min to collect the CTX‐TNA2 cell pellet. The harvested cells were then incubated with 5 μL fluorescein isothiocyanate (FITC)‐labeled Annexin V and 10 μL propidium iodide (PI) for 15 min at room temperature in the dark according to the manufacturer's protocol. After washing three times with PBS, all samples were analyzed by flow cytometry (Accuri C6 Plus; BD Biotechnology) to estimate the rate of apoptosis. At least 2 × 10^4^ cells were analyzed in each sample.

### 2.11 Western blotting (WB)

Proteins from the spinal cord on day 7 after SCI and cells were extracted with protease inhibitor cocktail PMSF (MB2678; Meilunbio), protein phosphatase inhibitor cocktail (P1260; Solarbio), and RIPA lysate (MA0151; Meilunbio). Protein concentrations were determined using a BCA kit (P0012S; Beyotime). Equal amounts of protein were separated by electrophoresis using FuturePAGE™ protein precast gel (ET12420LGel; ebio‐ace) for 1 h, transferred to NC membranes (66,485; Pall) using wet transfer method, incubated with 5% skimmed milk powder for 2 h at room temperature on shaking table. The membranes were then incubated with the primary antibody overnight at 4°C and finally incubated with the secondary antibody for 1 h at room temperature on a shaking table. The reaction bands were visualized using a hypersensitive ECL reagent (HY‐K1005; MedChemExpress) and exposed using a chemiluminescent imager (e‐BLOT). The density of protein bands was semi‐quantified using ImageJ (National Institutes of Health). The primary antibodies used in WB were as follows: rabbit anti‐C3 (1:2000, ab200999; Abcam), rabbit anti‐S100A10 (1:2000, PA5‐95505; Invitrogen), mouse anti‐β‐actin (1:10000, MA1‐140; Invitrogen), rabbit anti‐Caspase‐3 (1:2000, ab184787; Abcam), rabbit anti‐Bcl‐2 (1:1000, ab196495; Abcam), rabbit anti‐Bax (1:2000, ab32503; Abcam), rabbit anti‐GAPDH (1:2000, 2118; Cell Signaling Technology), rabbit anti‐JAK2 (1:5000, ab108596; Abcam), rabbit anti‐phospho‐JAK2 (1:2000, ab32101; Abcam), rabbit anti‐STAT3 (1:2000, ab68153; Abcam), and rabbit anti‐phospho‐STAT3 (1:5000, ab76315; Abcam). The secondary antibodies used in WB were as follows: Goat anti‐rabbit IgG H&L (HRP) (1:10000, SA00001‐2; Proteintech) and Goat anti‐mouse IgG H&L (HRP) (1:10000, G21040; Thermo Scientific).

### 2.12 Enzyme‐linked immunosorbent assay (ELISA)

The ELISA kits were used to evaluate the expression levels of anti‐inflammatory cytokines including TGF‐β (E‐EL‐R1015c; Elabscience) and IL‐10 (E‐EL‐R0016c; Elabscience) and pro‐inflammatory cytokines including TNF‐α (E‐EL‐R2856c; Elabscience) and IL‐1β (E‐EL‐R0012c; Elabscience) in the injured spinal cord tissue. On the 7th day after SCI, the hearts of rats were perfused with 0.9% normal saline, and then the spinal segments around the lesion center were removed. The spinal cord tissue was cut into 1 mm^3^ piece, and pre‐chilled RIPA protein lysate containing PMSF was added to grind into homogenates in liquid nitrogen. The nucleic acid was sonicated and lysed at 4°C for 20 min. The total protein was extracted by centrifugation at 12,000×*g* for 5 min. The supernatant was taken, and protein quantification was performed by BCA. The ELISA kits were used to detect the expression levels of TGF‐β and IL‐10 (anti‐inflammatory cytokines) and TNF‐α and IL‐1β (pro‐inflammatory cytokines) according to the manufacturer's protocol. The optical density values were measured with a microplate reader (Multiskan MK3; Thermo Fisher Scientific), and the corresponding cytokine concentrations were calculated from the standard curve.

### 2.13 Real‐time fluorescent quantitative PCR (qRT‐PCR)

Total RNA was extracted from cells and extracellular vesicles using TRIzol™ reagent (15,596,026; Invitrogen). Complementary DNA (cDNA) was then synthesized using an Applied Biosystems™ Veriti™ 96‐well fast thermal cycler. The synthesized cDNA was detected by qRT‐PCR using the SYBR Green method in a real‐time PCR instrument (ABI 7500; Thermo Fisher Scientific), and U6 was used as an internal reference for miRNA expression levels. Finally, the relative expression levels of miRNA were calculated using the 2^−ΔΔCT^ method. Primers miR‐21 and U6 were purchased from Shanghai Gemma Pharmaceutical Technology Co., Ltd., China. Primer sequences are listed in Table 1.

**TABLE 1:** qRT‐PCR Primer Sequences.

Primer	Sequences (5′–3′)
Rat‐miR‐21‐5p Forward	ACGTTGTGTAGCTTATCAGACTG
Rat‐miR‐21‐5p Reverse	AATGGTTGTTCTCCACACTCTC
U6 Forward	CAGCACATATACTAAAATTGGAACG
U6 Reverse	ACGAATTTGCGTGTCATCC

### 2.14 miRNAs detection in EV and H‐EV

The miRNA assays for EV and H‐EV were performed at Nanjing Jiangbei New District Biomedicine Public Service Platform Co., Ltd. (Nanjing, China) for three samples of each group. The miRNA sequencing analysis used the Illumina HiSeq 2000/2500 platform (Illumina). The *P* < 0.05 and | log2FoldChange | ≥ 1 standard was used to identify the differentially expressed miRNAs.

### 2.15 Statistical Analysis

All experiments were performed in at least three independent biological replicates. All data are shown as the mean ± standard deviation (SD). Graphic and statistical analyses were performed using GraphPad Software 8.0 (GraphPad Software). Shapiro–Wilk test was used to assess data distribution. All data conform to normality test. We used the two‐tailed student's *t* test to evaluate the statistical significance between the comparisons of two groups and one‐way ANOVA followed by Tukey's multiple comparison test to evaluate the statistical significance for multiple group comparisons. The BBB score was analyzed by two‐way ANOVA followed by Tukey's multiple comparison test to evaluate the statistical significance. *P* value <0.05 was considered statistically significant.

## 3 RESULTS

### 3.1 Identification of BMSCs

**FIGURE 1:**
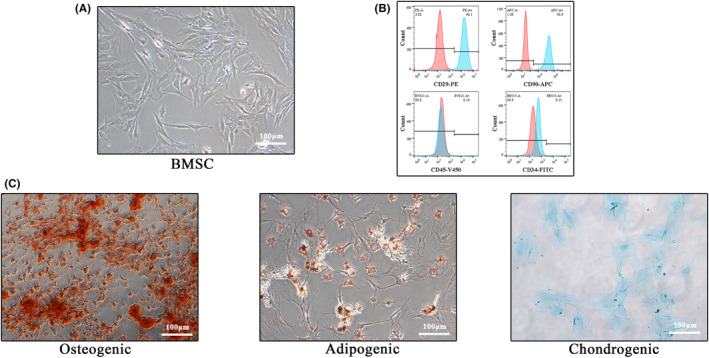
Identification of BMSCs. (A) Morphological image of BMSCs adherent growth under the light microscope. (B) Flow cytometric analysis of two positive (CD29 and CD90) and two negative surface protein markers (CD34 and CD45) of BMSCs. (C) Alizarin red, Orley red O, and Alcian blue staining were used to identify the osteogenic, lipogenic, and chondrogenic differentiation potential of BMSCs, respectively (Scale bar: 100 μm).

BMSCs were isolated from rat bone marrow as described above. In passage four, BMSCs were identified by morphology and flow cytometry. Cells were spindle‐shaped at 75–85% confluence (Figure 1A). Flow cytometry analysis (Figure 1B) was used to confirm that BMSCs were positive for CD29 and CD90 (>95%) but negative for CD34 and CD45 (<1%). Alizarin red, Orley red O, and Alcian blue staining were used to identify osteogenic, adipogenic, and chondrogenic differentiation of BMSCs, respectively (Figure 1C).

### 3.2 Identification of EV and H‐EV and astrocyte tracing in vitro

**FIGURE 2:**
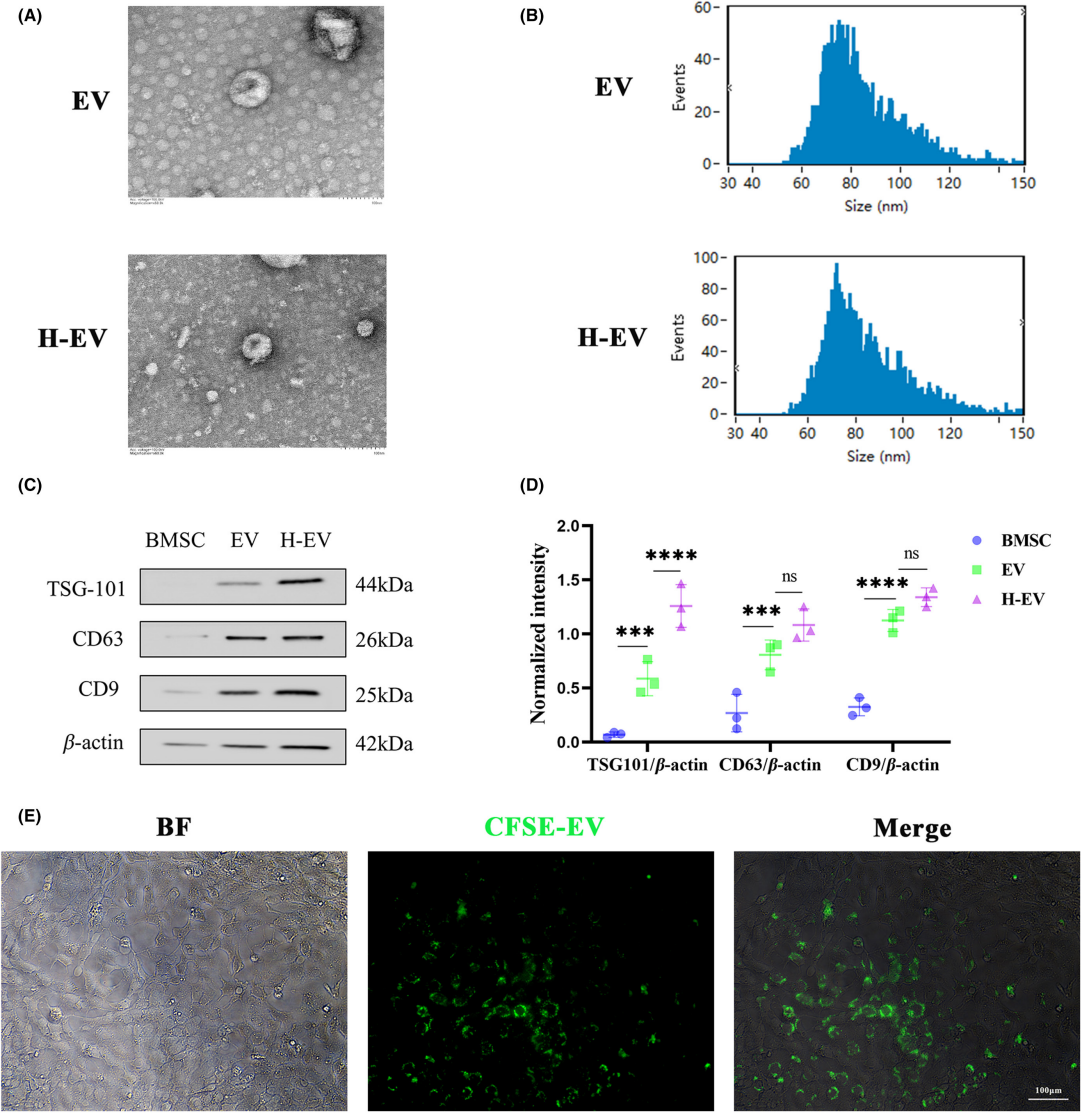
Identification of EV and H‐EV and astrocyte tracing in vitro. (A) Morphological images of EV and H‐EV under TEM. (B) Particle sizes of EV and H‐EV were analyzed by NTA. (C) Western blot analysis of three protein markers (TSG101, CD63, and CD9) of EV and H‐EV. (D) Quantitative analysis of TSG101, CD63, and CD9 protein expression levels relative to β‐Actin in (C), respectively (*n* = 3/group). (E) The uptake of CFSE‐labeled EV with green fluorescence by CTX‐TNA2 was observed under the fluorescence microscope (*P < 0.05, **P < 0.01, ***P < 0.001, ****P < 0.0001, ns: Non‐significant; Scale bar: 100 μm).

EV and H‐EV were isolated from an exosome‐free medium after incubation for 48 h under normoxic and hypoxic (1% O_2_) conditions using density gradient ultracentrifugation, respectively. They were then analyzed using TEM, NTA, and WB. TEM showed typical round nanoparticles (Figure 2A) with diameters between 30 and 150 nm, whereas NTA showed similar size distribution in normoxic and hypoxic groups (mean 84.12 nm vs. 84.05 nm) (Figure 2B). No morphological differences in size, shape, or electron density were observed between the two groups. WB showed the same surface markers including CD9, CD63, and TSG101 in normoxic and hypoxic groups (Figure 2C,D).

To check whether EVs were absorbed by CTX‐TNA2 astrocytes, BMSCs were labeled using CFSE dye, and then EVs were isolated and co‐cultured with CTX‐TNA2 for 24 h. Fluorescence microscopy was used to analyze the amount of EV uptake by CTX‐TNA2 astrocytes. The results showed that EV uptake by CTX‐TNA2 astrocytes was mainly distributed intracellularly rather than on the cell surface (Figure 2E).

### 3.3 EV and H‐EV promote functional recovery after SCI in vivo

**FIGURE 3:**
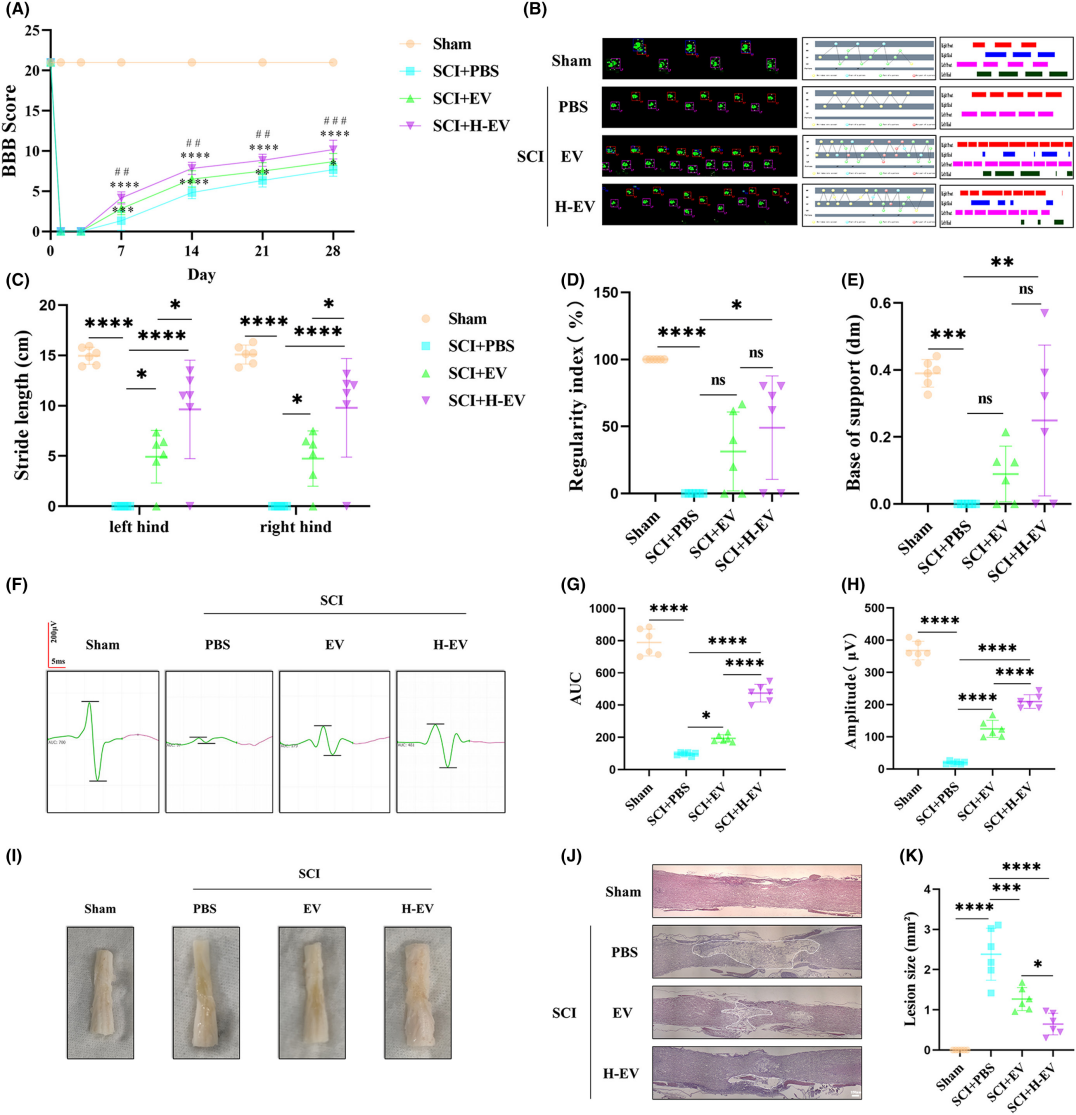
EV and H‐EV promote functional recovery after SCI in vivo. (A) BBB score of sham, SCI + PBS, SCI + EV, and SCI + H‐EV groups on days 1, 3, 7, 14, 21, and 28 (*n* = 6/group). (B) Representative Catwalk footprint analysis of sham, SCI + PBS, SCI + EV, and SCI + H‐EV groups at day 28 (*n* = 6/group). (C–E) Three parameters of footprint analysis were used to quantify the motion function. (C) Stride length. (D) Regularity index. (E) The base of support. (F) Representative MEP analysis of sham, SCI + PBS, SCI + EV, and SCI + H‐EV groups at day 28 (*n* = 6/group). (G–H) Two parameters of MEP analysis were used to quantify the muscle strength of hind limbs. (G) AUC. (H) Amplitude. (I) Representative the gross morphology of the spinal cord of sham, SCI + PBS, SCI + EV, and SCI + H‐EV groups at day 28 (*n* = 6/group). (J) Representative the HE staining of the spinal cord of sham, SCI + PBS, SCI + EV, and SCI + H‐EV groups at day 28 (*n* = 6/group). (K) The size of the lesion was quantified by HE staining (*n* = 6/group) (*P < 0.05, **P < 0.01, ***P < 0.001, ****P < 0.0001, vs SCI + PBS; #*P* < 0.05, ##*P* < 0.01, ###*P* < 0.001, ####*P* < 0.0001, vs SCI + EV; ns: non‐significant; scale bar: 250 μm).

In this study, to investigate whether H‐EV could have a more beneficial effect on motor function after SCI than EV, we first evaluated the functional recovery of rats in sham, SCI + PBS, SCI + EV, and SCI + H‐EV groups using the BBB score (Figure 3A). Compared to rats in the PBS group, rats in the EV group showed better functional improvement, which is consistent with many previous studies.^51^, ^52^ However, in our study, we noticed a significant increase in BBB score in the H‐EV group compared to the EV group. The results of the footprint analysis were also consistent with the BBB score (Figure 3B). At day 28 after SCI, rats treated with EV showed significantly faster gait recovery and improved motor coordination compared with rats in the PBS group, and this favorable effect was more pronounced in the H‐EV group. This result was observed from the stride lengths of the left and right hind limbs (Figure 3C). However, neither the normal step ratio nor the hind limb support width metrics were statistically significant in the PBS and H‐EV groups compared with the EV group (Figure 3D,E). However, both the normal step ratio and hind limb support width metrics were statistically significant in the H‐EV group compared with the control group (Figure 3D,E), indicating that H‐EV treatment contributed to motor function recovery in SCI rats. To further investigate behavioral recovery of motor function, MEP analysis was applied as the electrophysiological analysis (Figure 3F). At day 28 post‐injury, the H‐EV group showed higher AUC (Figure 3G) and amplitude (Figure 3H) than the EV group, indicating that the hind limb in the H‐EV group showed better electrophysiological functional recovery than the EV group. The traumatic lesion site was visible in the gross morphology of the injured spinal cord (Figure 3I), and the lesion area in the EV and H‐EV groups was significantly smaller than in the PBS group. These results also showed that the H‐EV group had significantly smaller lesion areas than the EV group. HE staining was also consistent with gross morphology (Figure 3J,K). Compared with the PBS group, the EV group showed a significantly smaller lesion area. However, the H‐EV group showed a smaller lesion area than the EV group, indicating that the H‐EV group was more effective than the EV group in the lesion area. In conclusion, these results suggest that both EV and H‐EV administered intravenously can promote functional behavioral recovery after SCI in rats, and these beneficial effects in the H‐EV group are more pronounced than in the EV group.

### 3.4 EV and H‐EV play a role in promoting astrocyte conversion from A1 to A2 in vivo

**FIGURE 4:**
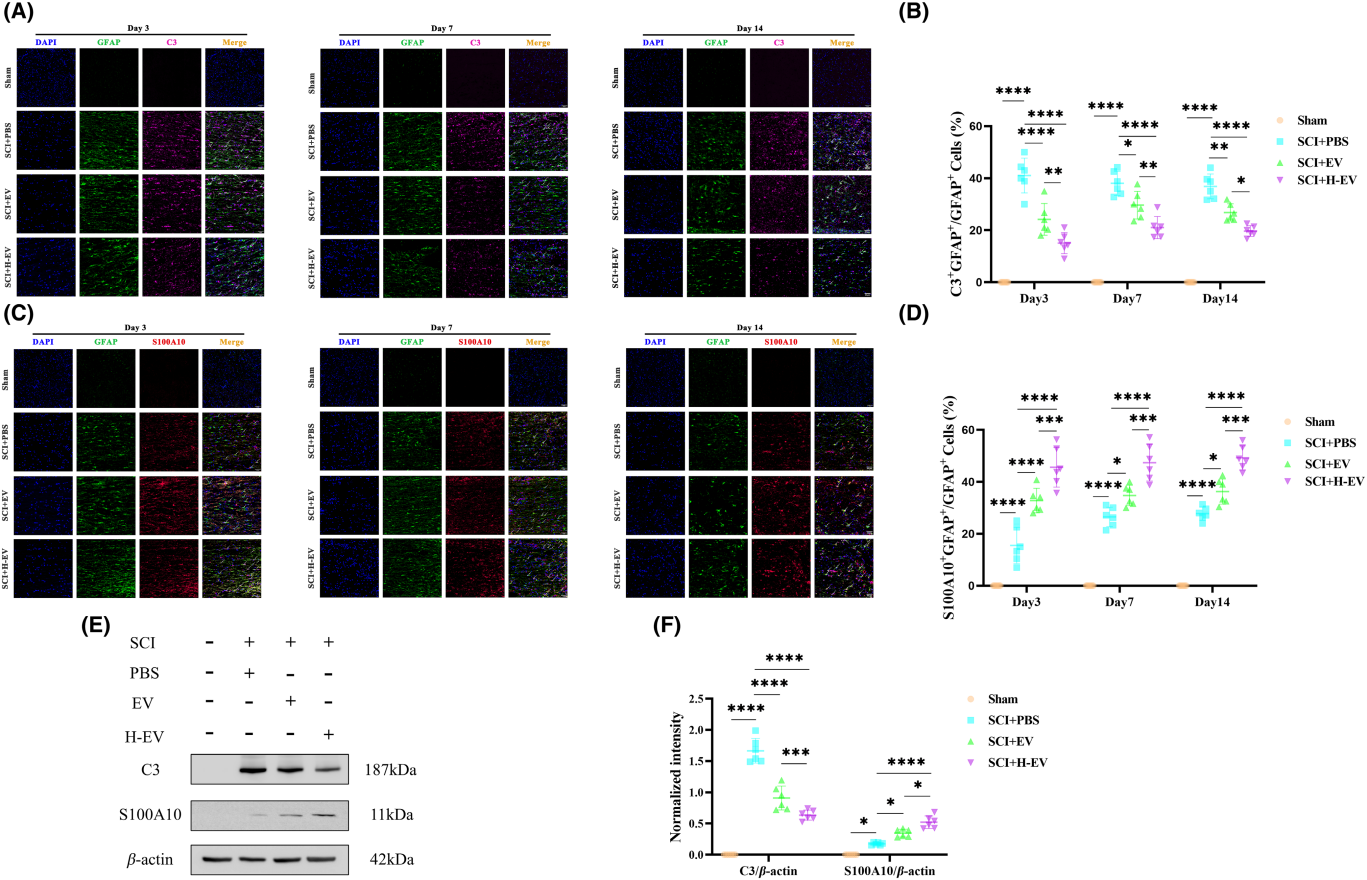
EV and H‐EV play a role in promoting astrocyte conversion from A1 to A2 in vivo. (A) Representative the immunofluorescence images of A1 astrocytes of the spinal cord in sham, SCI + PBS, SCI + EV, and SCI + H‐EV groups on days 3, 7, and 14 (*n* = 6/group, DAPI: Blue, GFAP: Green, C3: Far red, white arrow: C3^+^GFAP^+^ A1 astrocytes). (B) Quantitative analysis of C3^+^GFAP^+^/GFAP^+^ in (A). (C) Representative the immunofluorescence images of A2 astrocytes of the spinal cord in sham, SCI + PBS, SCI + EV, and SCI + H‐EV groups at day 3, 7, and 14 (*n* = 6/group, DAPI (blue), GFAP (green), S100A10 (red), white arrow: S100A10^+^GFAP^+^ A2 astrocytes). (D) Quantitative analysis of S100A10^+^GFAP^+^/GFAP^+^ in (C). (E) Representative western blot analysis of the expression levels of three protein markers of the spinal cord in sham, SCI + PBS, SCI + EV, and SCI + H‐EV groups at day 7 (*n* = 6/group, C3: A1 Astrocyte, S100A10: A2 Astrocyte, β‐Actin: Internal reference protein). (F) Quantitative analysis of C3 and S100A10 expression levels relative to β‐Actin in (E), respectively (*P < 0.05, **P < 0.01, ***P < 0.001, ****P < 0.0001, scale bar: 50 μm).

Since astrocytes can have two distinct phenotypes, we questioned whether the administration of EV and H‐EV could polarize astrocytes from A1 to A2 phenotype after SCI. On days 3, 7, and 14 after SCI, we evaluated the characteristic polarization of astrocytes in different groups after SCI using representative A1‐related C3 and A2‐related S100A10 markers together with GFAP, GFAP which can detect astrocytes in the injured spinal cord. The immunofluorescence results of the spinal cord showed that the number of C3^+^GFAP^+^ cells was significantly increased (Figure 4A,B) and the number of S100A10^+^GFAP^+^ cells was significantly decreased (Figure 4C,D) in the PBS group compared with the sham group. Compared with the PBS group, the number of C3^+^GFAP^+^ cells was significantly decreased (Figure 4A,B), and the number of S100A10^+^GFAP^+^ cells was significantly increased (Figure 4C,D) in the EV and H‐EV groups. Interestingly, the number of C3^+^GFAP^+^ cells tended to be lower in the H‐EV group compared to the EV group (Figure 4A,B), while the number of S100A10^+^GFAP^+^ cells in the H‐EV group was higher than the EV group (Figure 4C,D). This result indicated that the H‐EV treatment had a more significant effect than the EV treatment on the A1/2 polarization of astrocytes. To further demonstrate our results, we selected the SCI rats with relatively high expression levels of C3 and S100A100 at day 7. After total protein extraction, WB was used to detect the protein expression levels, which showed the same trend as the results of immunofluorescence (Figure 4E,F). In conclusion, both EV and H‐EV promoted astrocyte transformation from A1 to A2 phenotype, and the promoting effect of H‐EV was more pronounced than that of EV.

### 3.5 EV and H‐EV play a role in anti‐inflammatory and anti‐apoptotic effects in vivo

**FIGURE 5:**
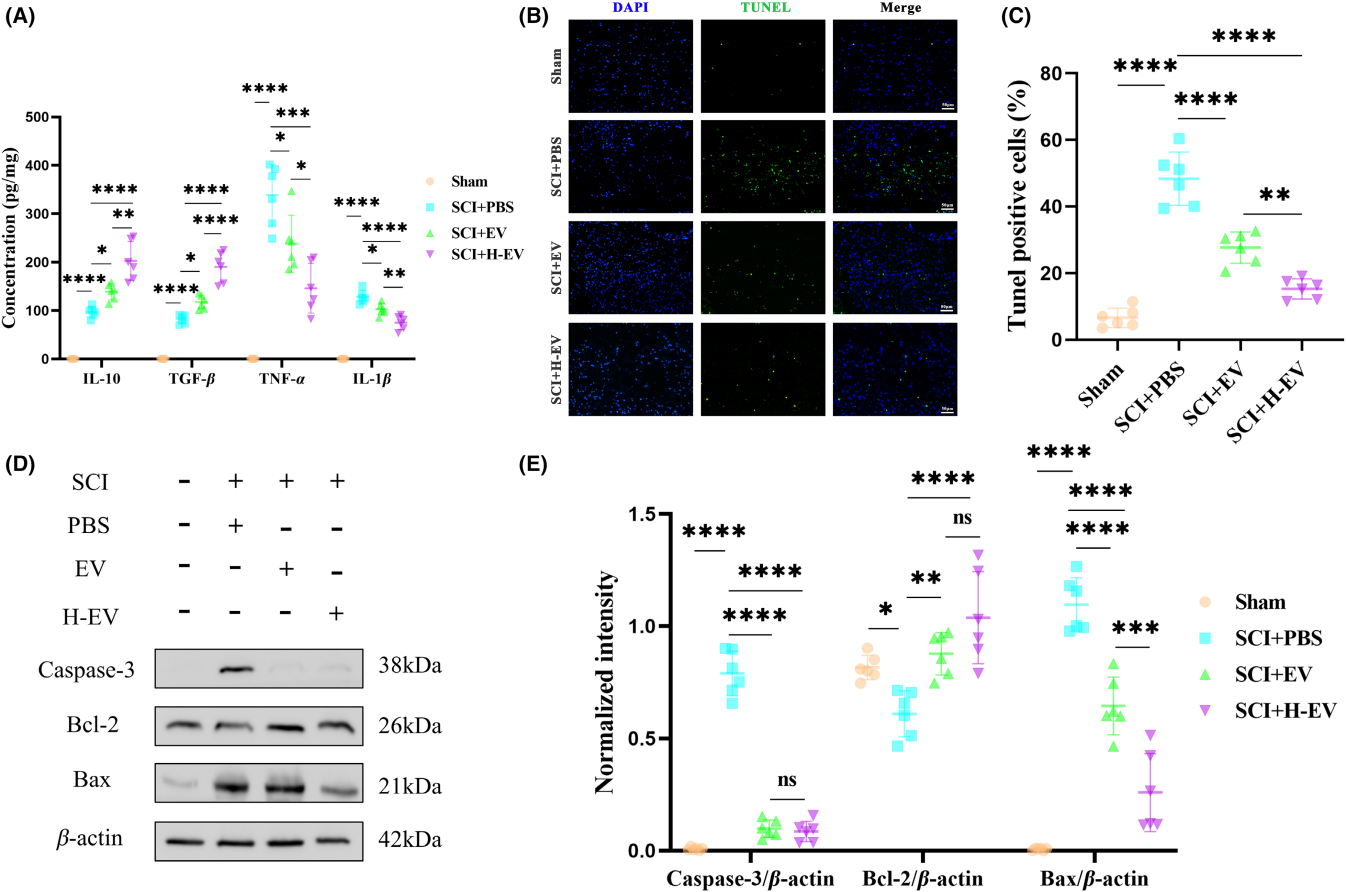
EV and H‐EV play a role in anti‐inflammatory and anti‐apoptotic effects in vivo. (A) ELISA was used to measure the concentrations of pro‐inflammatory cytokines (TNF‐α and IL‐1β) and anti‐inflammatory cytokines (TGF‐β and IL‐10) of the spinal cord in sham, SCI + PBS, SCI + EV, and SCI + H‐EV groups at day 7 (*n* = 6/group). (B) Representative the immunofluorescence images of TUNEL staining in sham, SCI + PBS, SCI + EV, and SCI + H‐EV groups at day 7 (*n* = 6/group, DAPI: Blue, TUNEL: Green, TUNEL‐positive cells: Apoptotic nerve cells). (C) Quantitative analysis of TUNEL‐positive cells as a percentage of the total number of cells in (B). (D) Representative western blot analysis of the expression levels of pro‐apoptotic proteins (Bax and Caspase‐3) and anti‐apoptotic protein (Bcl‐2) of the spinal cord in sham, SCI + PBS, SCI + EV, and SCI + H‐EV groups at day 7 (*n* = 6/group). (E) Quantitative analysis of Caspase‐3, Bcl‐2, and Bax protein expression levels relative to β‐Actin in (D), respectively (*P < 0.05, **P < 0.01, ***P < 0.001, ****P < 0.0001, ns: Non‐significant, scale bar: 50 μm).

At the same time, we also detected the same samples by ELISA to measure the concentrations of pro‐inflammatory cytokines (TNF‐α and IL‐1β) and anti‐inflammatory cytokines (TGF‐β and IL‐10) in spinal cord tissues in different groups. The results indicated that compared to the sham group, both pro‐inflammatory cytokines (TNF‐α and IL‐1β) and anti‐inflammatory cytokines (TGF‐β and IL‐10) were significantly increased in the PBS group. These results also showed that pro‐inflammatory cytokines (TNF‐α and IL‐1β) were significantly decreased, while anti‐inflammatory cytokines (TGF‐β and IL‐10) were significantly increased in the EV and H‐EV groups compared with the PBS group. However, treatment with H‐EV greatly promoted the secretion of anti‐inflammatory cytokines (TGF‐β and IL‐10) and suppressed the secretion of pro‐inflammatory cytokines (TNF‐α and IL‐1β) compared with the EV treatment (Figure 5A). Thus, these results indicate that both EV and H‐EV have a significant impact on the ratio of anti‐inflammatory to pro‐inflammatory phenotypes after SCI, and can shift astrocyte polarization from A1 to A2 phenotype. Even more, the H‐EV group showed better efficacy compared to the EV group. Finally, on day 7 after SCI, we used the Tunel method to detect the cell apoptosis in the spinal cord tissue, and immunofluorescence results showed that the number of Tunel‐positive cells was markedly increased in the PBS group compared with the sham group. The number of Tunel‐positive cells was decreased in the EV and H‐EV groups compared with the PBS group. However, the number of Tunel‐positive cells in the H‐EV group was lower than in the EV group (Figure 5B,C). In addition, we also used WB to detect the expression levels of pro‐apoptotic proteins (Bax and Caspase‐3) and anti‐apoptotic protein (Bcl‐2) in the spinal cord tissue. The results showed that the PBS group showed a significant increase in pro‐apoptotic proteins (Bax and Caspase‐3) and a significant decrease in anti‐apoptotic protein (Bcl‐2) compared to the sham group. Both EV and H‐EV treatments significantly decreased the expression level of pro‐apoptotic proteins (Bax and Caspase‐3) and increased the expression level of anti‐apoptotic protein (Bcl‐2) compared to the PBS group. However, treatment with H‐EV greatly promoted the expression of the anti‐apoptotic protein (Bcl‐2) and suppressed the expression of pro‐apoptotic proteins (Bax and Caspase‐3) compared with the EV treatment (Figure 5D,E). In conclusion, these results suggest that both EV and H‐EV administered intravenously promote anti‐inflammatory and anti‐apoptotic effects after SCI in rats, and these beneficial effects in the H‐EV group are more pronounced than in the EV group.

### 3.6 EV and H‐EV promote astrocyte phenotype switching from A1 to A2 and anti‐apoptotic effect in vitro

**FIGURE 6:**
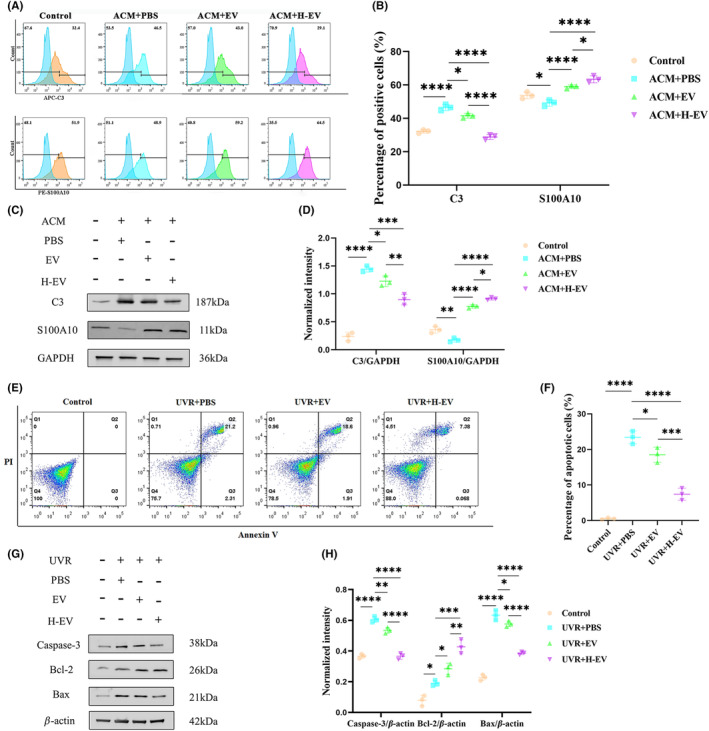
EV and H‐EV promote astrocyte phenotype switching from A1 to A2 and anti‐apoptotic effect in vitro. (A) Representative flow cytometric analysis of A1‐related C3 and A2‐related S100A10 markers of CTX‐TNA2 astrocytes in control, ACM + PBS, ACM + EV, and ACM + H‐EV groups (*n* = 3/group). (B) Quantitative analysis of C3 positive and S100A10 positive cells as a percentage of the total number of CTX‐TNA2 astrocytes in (A), respectively. (C) Representative western blot analysis of the expression levels of CTX‐TNA2 astrocytes A1‐related C3 and A2‐related S100A10 protein markers in control, ACM + PBS, ACM + EV, and ACM + H‐EV groups (*n* = 3/group, GAPDH: Internal reference protein). (D) Quantitative analysis of C3 and S100A10 protein expression levels relative to GAPDH in (C), respectively. (E) Representative the flow cytometric analysis of Annexin V‐FITC/PI double staining of CTX‐TNA2 astrocytes in control, UVR + PBS, UVR + EV, and UVR + H‐EV groups (*n* = 3/group, both Annexin V and PI positive cells in the Q2 quadrant: Apoptotic cells). (F) Quantitative analysis of apoptotic cells as a percentage of the total number of cells in (E). (G) Representative western blot analysis of the expression levels of pro‐apoptotic proteins (Bax and Caspase‐3) and anti‐apoptotic protein (Bcl‐2) of CTX‐TNA2 astrocytes in control, UVR + PBS, UVR + EV, and UVR + H‐EV groups (*n* = 3/group). (H) Quantitative analysis of Caspase‐3, Bcl‐2, and Bax protein expression levels relative to β‐Actin in (G), respectively (*P < 0.05, **P < 0.01, ***P < 0.001, ****P < 0.0001).

To determine whether EV and H‐EV exert therapeutic effects similar to those observed in vivo affecting phenotypic changes in astrocytes, ACM was first added to the culture system for 24 h to induce an inflammatory microenvironment to mimic SCI in vivo and then ACM was washed off and co‐cultured with PBS, EV, or H‐EV for 24 h. Cells were collected, and cellular proteins were extracted to observe the therapeutic effect. We used flow cytometry to analyze the altered phenotype of CTX‐TNA2 astrocytes, and the results showed that the number of C3^+^ cells was significantly increased and the number of S100A10^+^ cells was significantly decreased in the PBS group compared with the control group. Both EV and H‐EV treatments significantly decreased the number of C3^+^ cells and increased the number of S100A10^+^ cells compared to the PBS group. Interestingly, the number of C3^+^ cells in the H‐EV group tended to be lower compared to the EV group, whereas the number of S100A10^+^ cells in the H‐EV group was higher than in the EV group. This result highlights that H‐EV treatment contributes more to astrocyte conversion from A1 to A2 than EV treatment (Figure 6A,B). The results of WB also confirmed that of flow cytometry (Figure 6C,D). In conclusion, these results suggest that H‐EV play a more prominent role than EV in shifting the CTX‐TNA2 astrocyte phenotype from A1 to A2 in vitro, which is consistent with the results observed in vivo.

Similarly, in vitro, to determine whether the anti‐apoptotic effects of EV and H‐EV were similar to results in vivo, we irradiated CTX‐TNA2 astrocytes with ultraviolet light (UVR) for 2 h to mimic the apoptosis model, as described previously, and then co‐cultured with PBS, EV, or H‐EV for 24 h. The cells and their proteins were collected to observe the therapeutic effect. We used Annexin V‐FITC/PI double staining to detect apoptosis of CTX‐TNA2 astrocytes, and flow cytometry results showed that the number of apoptotic cells in the PBS group was significantly increased compared with the control group. EV and H‐EV groups showed decreased numbers of apoptotic cells compared to the PBS group. However, there were fewer apoptotic cells in the H‐EV group than in the EV group (Figure 6E,F). In addition, we also used WB to detect the expression levels of pro‐apoptotic proteins (Bax and Caspase‐3) and anti‐apoptotic protein (Bcl‐2) in CTX‐TNA2 astrocytes. The results showed that the PBS group showed a significant increase in pro‐apoptotic proteins (Bax and Caspase‐3) and a significant decrease in anti‐apoptotic protein (Bcl‐2) compared to the control group. Both EV and H‐EV treatments significantly decreased the expression level of pro‐apoptotic proteins (Bax and Caspase‐3) and increased the expression level of anti‐apoptotic protein (Bcl‐2) compared to the PBS group. However, treatment with H‐EV greatly promoted the expression of the anti‐apoptotic protein (Bcl‐2) and suppressed the expression of pro‐apoptotic proteins (Bax and Caspase‐3) compared with EV (Figure 6G,H). These results are consistent with those observed in vivo, and both suggest that H‐EV can inhibit apoptosis more than EV.

### 3.7 miR‐21 is up‐regulated in H‐EV and H‐EV promotes astrocyte transformation from A1 to A2 by transporting miR‐21

**FIGURE 7:**
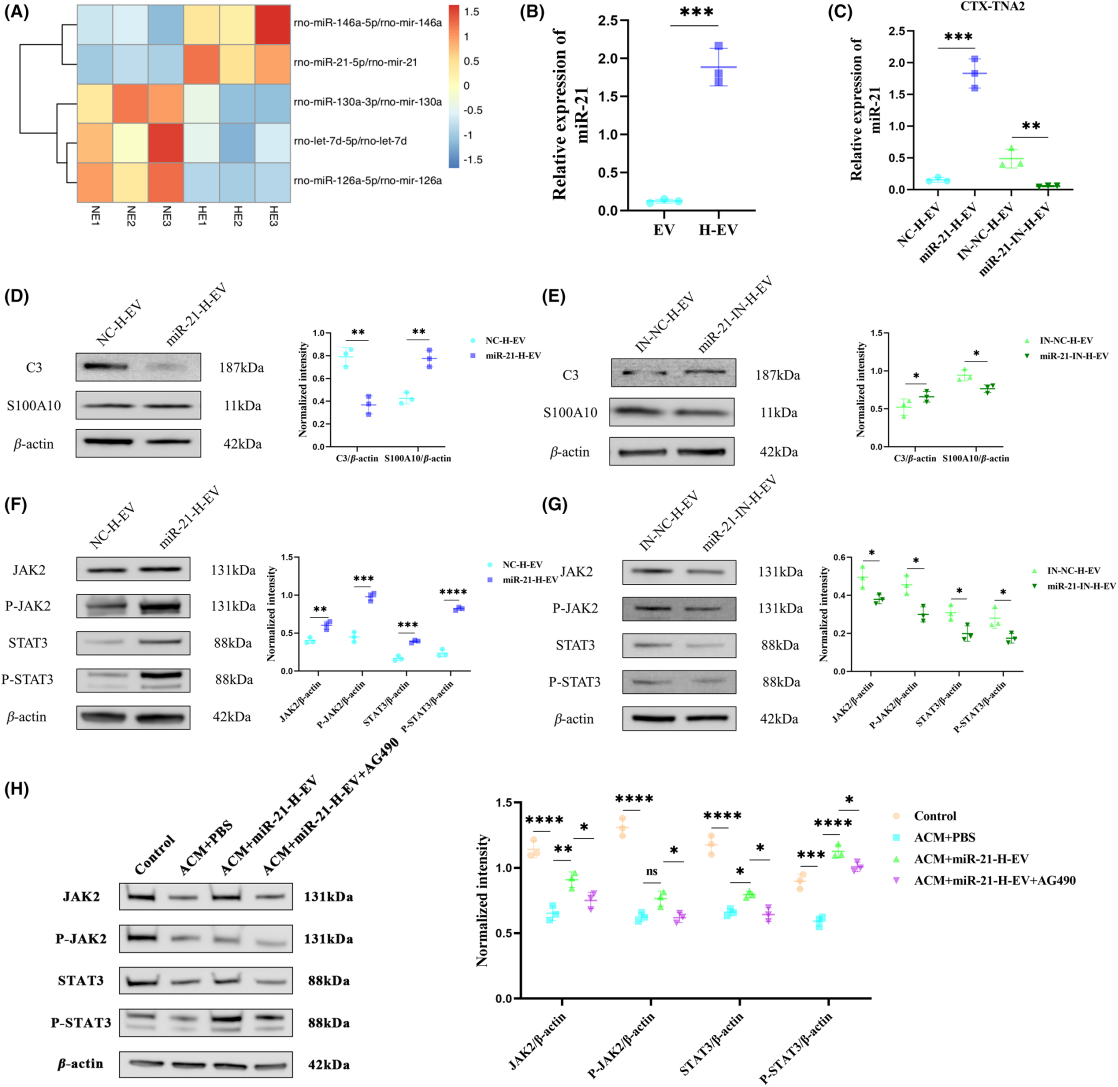
Mir‐21 is up‐regulated in H‐EV and H‐EV promote astrocyte transformation from A1 to A2 via miR‐21/JAK2/STAT3 pathway. (A) Heat map of the two up‐regulated and three down‐regulated miRNAs between EV and H‐EV (*n* = 3/group, the *P* < 0.05 and |log2FoldChange| ≥ 1 standard). (B) qRT‐PCR was used to verify the difference in miR‐21 expression levels between EV and H‐EV (*n* = 3/group). (C) qRT‐PCR was used to verify the difference in miR‐21 expression levels of CTX‐TNA2 astrocytes in NC‐H‐EV, miR‐21‐H‐EV, IN‐NC‐H‐EV, and miR‐21‐IN‐H‐EV groups (*n* = 3/group). (D) Representative western blot analysis and relative quantitative of the expression levels of CTX‐TNA2 astrocytes A1‐related C3 and A2‐related S100A10 protein markers in NC‐H‐EV and miR‐21‐H‐EV groups (*n* = 3/group, β‐Actin: Internal reference protein). (E) Representative western blot analysis and relative quantitative of the expression levels of CTX‐TNA2 astrocytes A1‐related C3 and A2‐related S100A10 protein markers in IN‐NC‐H‐EV and miR‐21‐IN‐H‐EV groups (*n* = 3/group, β‐Actin: Internal reference protein). (F) Representative western blot analysis and relative quantitative of the expression levels of JAK2, P‐JAK2, STAT3, and P‐STAT3 proteins in NC‐H‐EV and miR‐21‐H‐EV groups (*n* = 3/group, β‐Actin: Internal reference protein). (G) Representative western blot analysis and relative quantitative of the expression levels of JAK2, P‐JAK2, STAT3, and P‐STAT3 proteins in IN‐NC‐H‐EV and miR‐21‐IN‐H‐EV groups (*n* = 3/group, β‐Actin: Internal reference protein). (H) Representative western blot analysis and relative quantitative of the expression levels of JAK2, P‐JAK2, STAT3, and P‐STAT3 proteins in control, ACM + PBS, ACM + miR‐21‐H‐EV, and ACM + miR‐21‐H‐EV + AG490 groups (*n* = 3/group, β‐Actin: Internal reference protein) (*P < 0.05, **P < 0.01, ***P < 0.001, ****P < 0.0001).

We compared the efficacy of EV and H‐EV in vitro and in vivo and found that H‐EV was superior to EV in terms of motor function, CTX‐TNA2 astrocyte polarization, apoptosis, and inflammatory response. Since many studies have found that miRNA plays an important role in its efficacy,^35^, ^36^, ^37^ therefore, we performed miRNA sequencing on EV and H‐EV and found that two miRNAs were up‐regulated (miR‐146a and miR‐21) and three miRNAs were down‐regulated (miR‐130a, let‐7d, and miR‐126a) in H‐EV compared to EV (Figure 7A). Then, we selected the most significantly up‐regulated miR‐21 to verify whether it could alter CTX‐TNA2 astrocyte polarization. First, we verified with qRT‐PCR that it was indeed up‐regulated in H‐EV (Figure 7B). Then, we incubated miR‐21 mimics and inhibitors and their corresponding negative controls with transfection reagents and H‐EV for the treatment of astrocytes after ACM stimulation. Total RNA was extracted from CTX‐TNA2 astrocytes, and qRT‐PCR was used to verify the expression level of miR‐21 in astrocytes. The results showed that the expression level of miR‐21 in the miR‐21‐H‐EV group was significantly higher than that in the NC‐H‐EV group. However, the expression level of miR‐21 in the miR‐21‐IN‐H‐EV group was significantly lower than that in the IN‐NC‐H‐EV group (Figure 7C). Meanwhile, we assessed the effect of miR‐21 on CTX‐TNA2 astrocyte phenotypic alterations in vitro. We detected the expression levels of C3 and S100A10 in CTX‐TNA2 astrocytes by WB, and the results showed that the expression level of C3 was significantly decreased and the expression level of S100A10 was significantly increased in the miR‐21‐H‐EV group compared with the NC‐H‐EV group (Figure 7D). However, compared with the IN‐NC‐H‐EV group, the expression level of C3 was significantly increased and the expression level of S100A10 was significantly decreased in the miR‐21‐IN‐H‐EV group (Figure 7E). These results indicated that H‐EV could play an important role in switching the phenotype of CTX‐TNA2 astrocytes from A1 to A2 in vitro through miR‐21.

### 3.8 miR‐21 within H‐EV transforms astrocytes from A1 to A2 via JAK2/STAT3 pathway

It has been shown that the JAK2/STAT3 pathway plays an important role in regulating astrocyte phenotype change, and the increase of the JAK2/STAT3 expression level is conducive to the transformation of the astrocyte phenotype from A1 to A2.^41^ Based on this finding, we detected the expression levels of JAK2, P‐JAK2, STAT3, and P‐STAT3 in the four groups of NC‐H‐EV, miR‐21‐H‐EV, IN‐NC‐H‐EV, and miR‐21‐IN‐H‐EV by WB. The results showed that compared with the NC‐H‐EV group, the expression levels of JAK2, P‐JAK2, STAT3, and P‐STAT3 were significantly increased in the miR‐21‐H‐EV group (Figure 7F). However, the expression levels of JAK2, P‐JAK2, STAT3, and P‐STAT3 were significantly decreased in the miR‐21‐IN‐H‐EV group compared with the IN‐NC‐H‐EV group (Figure 7G). These results indicated that miR‐21 within H‐EV may affect the phenotypic changes of astrocytes through JAK2/STAT3 pathway. In order to further validate the targeted regulation of JAK2/STAT3 by miR‐21, we conducted experiments using AG490, a specific inhibitor of JAK2/STAT3. The results revealed a significant decrease in the expression levels of JAK2, P‐JAK2, STAT3, and P‐STAT3 compared to the control group after ACM stimulation (Figure 7H). Moreover, when compared to the PBS untreated group, treatment with H‐EV overexpressing miR‐21 led to a significant increase in the expression levels of JAK2, P‐JAK2, STAT3, and P‐STAT3 (Figure 7H). Importantly, the ability of miR‐21 in H‐EV to enhance the expression levels of JAK2, P‐JAK2, STAT3, and P‐STAT3 was counteracted when AG490 was simultaneously administered (Figure 7H). These findings provide clear evidence of the regulatory relationship between miR‐21 and JAK2/STAT3, demonstrating that miR‐21 can target the JAK2/STAT3 signaling pathway and regulate their expression levels.

### 3.9 miR‐21 within H‐EV enhances functional recovery and induces astrocyte phenotype switching from A1 to A2 after SCI in vivo

We have demonstrated the important role of miR‐21 in astrocyte phenotypic changes in H‐EV in vitro. In order to further explore whether miR‐21 in H‐EV also plays a significant role in SCI rats, we administered miR‐21 up agomir, agomir NC, miR‐21 down antagomir, and antagomir NC via intravenous injection while simultaneously providing H‐EV treatment. The results of the BBB scoring indicated that compared to the agomir NC group, the miR‐21 up agomir group had a higher BBB score (Figure S1A). However, compared to the antagomir NC group, the miR‐21 down antagomir group had a lower BBB score (Figure S1A). These results suggest that miR‐21 can promote motor function recovery in SCI rats. Additionally, on day 28 after SCI, we performed catwalk gait analysis on the rats (Figure S1B). The results of stride length, regularity index, and the base of support demonstrated that the miR‐21 up agomir group had better therapeutic effects compared to the agomir NC group (Figure S1C–E). However, compared to the antagomir NC group, the miR‐21 down antagomir group showed poorer therapeutic effects (Figure S1C–E). These catwalk gait analysis results were highly consistent with the BBB scoring results, providing strong evidence for the crucial role of miR‐21 in SCI rats. Furthermore, we used WB analysis to assess astrocyte phenotypic changes in the spinal cord tissue of SCI rats. The results showed that compared to the agomir NC group, the miR‐21 up agomir group exhibited significantly reduced C3 expression and increased S100A10 expression (Figure S1). However, compared to the antagomir NC group, the miR‐21 down antagomir group showed significantly increased C3 expression and decreased S100A10 expression (Figure S1G). These findings confirm that miR‐21 indeed promotes the transition of astrocytes from the A1 phenotype to the A2 phenotype in SCI rats.

## 4 DISCUSSION

**FIGURE 8:**
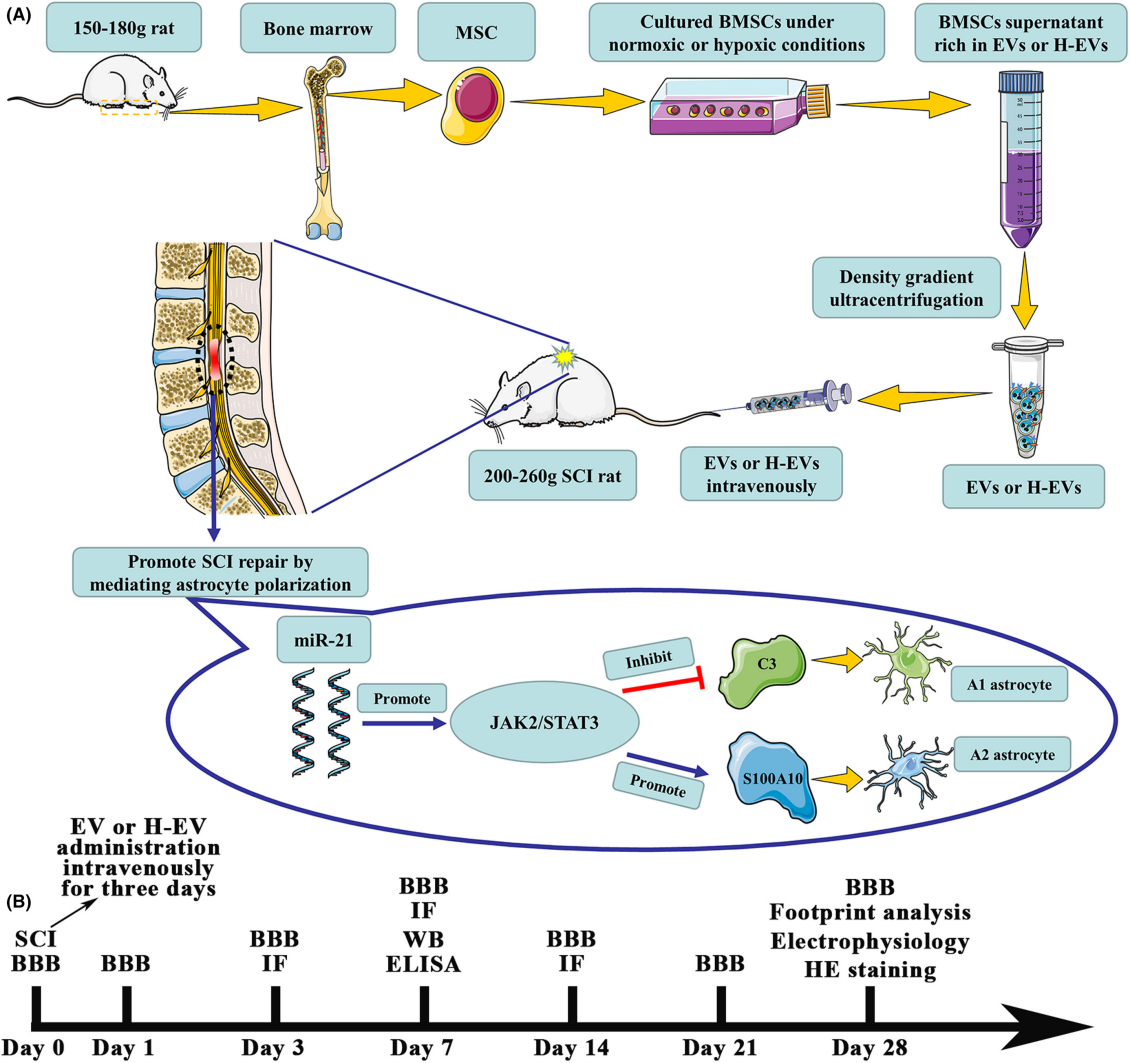
H‐EV promotes SCI repair by affecting the phenotype of astrocytes through the miR‐21/JAK2/STAT3 pathway. (A) Schematic diagram of the experimental process. (B) The time chart of experiments in vivo.

This study showed that both EV and H‐EV administered via the tail vein contributed to the recovery of motor function in SCI rats, and H‐EV was more effective than the EV. We also demonstrated that H‐EV was superior to EV in terms of apoptosis and inflammatory response after SCI. Our focus was on the altered astrocyte phenotypes after SCI. It was found that both EV and H‐EV could promote the transformation of astrocytes from A1 to A2 phenotype. More importantly, the effect of H‐EV on promoting phenotypic changes was more obvious than that of EV. To further explore the reasons for the difference in efficacy between EV and H‐EV, we found that many studies focused on the contents of proteins and RNAs of EVs, among which miRNAs may play an important role. By sequencing the miRNAs of EV and H‐EV, we found a significant increase in miR‐21 in H‐EV compared to EV. Therefore, miR‐21 was selected to further verify its mechanism of action. The effect of knockdown and overexpression of miR‐21 in H‐EV on phenotypic changes of astrocytes was observed, and it was found that the expression level of C3 was significantly decreased and the expression level of S100A10 was significantly increased when the miR‐21 was overexpressed by H‐EV for the treatment of astrocytes in the inflammatory state compared with the control group. However, the expression level of C3 was significantly increased, and the expression level of S100A10 was significantly decreased when the miR‐21 was knocked down by H‐EV for the treatment of astrocytes in an inflammatory state compared with the control group. This result suggested that H‐EV promoted the transformation of astrocytes from A1 to A2 phenotype by delivering miR‐21, thereby promoting the repair of SCI. To further investigate its mechanism of action and target, we performed WB of JAK2, P‐JAK2, STAT3, and P‐STAT3, and showed that the expression levels of JAK2, P‐JAK2, STAT3, and P‐STAT3 were significantly increased in the H‐EV group with overexpression of miR‐21 compared with the control group. Compared with the control group, the expression levels of JAK2, P‐JAK2, STAT3, and P‐STAT3 were significantly decreased in the H‐EV group with a knockdown of miR‐21. The trend was consistent with the expression level of miR‐21, indicating that H‐EV may promote the transformation of type A1 astrocytes into type A2 astrocytes through the miR‐21/JAK2/STAT3 pathway, thus playing a neuroprotective role and ultimately promoting the repair of SCI (Figure 8A,B).

Clinically, the treatment of patients with SCI in the acute phase is mainly through timely surgical decompression to relieve compression and reduce serious secondary injury, which shows that the inflammatory cascade caused by secondary injury cannot be ignored.^6^ For neuroinflammatory responses after SCI, most studies focus on the mechanism of action and treatment of microglia or macrophages but ignore intercellular communication.^53^ Recent studies have shown that astrocytes, as abundant cells in the central nervous system, also play a crucial role in the regulation of neuroinflammation.^12^, ^54^ In the mouse model of LPS‐induced inflammatory response, it was found that astrocytes were activated by cytokines (IL‐1α, TNF‐α, and C1q) released from LPS‐induced microglia and turned into A1 astrocytes, which secreted toxic substances and promoted inflammatory response.^15^ Meanwhile, in the mouse model of ischemia and hypoxia induced by middle cerebral artery occlusion, astrocytes were also induced to differentiate into the A2 phenotype, which secreted serious neurotrophic factors and inhibited the inflammatory response, playing a neuroprotective role.^16^ Homer scaffold protein 1 (Homer1), as a neuroprotective protein, was found to improve the functional recovery of intracerebral hemorrhage in mice by promoting the transformation of A1 astrocytes into A2 astrocytes.^46^ Therefore, inhibiting the transformation of astrocytes from A1 to A2 may provide new ideas and great potential for the regulation of neuroinflammation.

EVs are widely studied intercellular communication tools, which contain various lipids, proteins, RNA, and DNA.^55^, ^56^ In recent years, many studies have shown that MSC‐EVs can exert the same function as MSCs and are widely used in the study of the treatment of diseases in various fields.^57^, ^58^ More importantly, they can make up for the deficiency of stem cells and have low immunogenicity and tumorigenicity.^59^ In the field of the central nervous system, it has been found that EV can overcome the obstacles of the blood–brain barrier, and intravenous administration after SCI can enrich the injured site and exert a direct effect.^24^ However, because it is difficult for cells to penetrate the blood–brain barrier to reach sites of central nervous system injury, the advantages of EV will make it expected to replace stem cells as a new therapeutic star in some fields.^60^ In recent years, it has been shown that MSC‐EVs can promote the repair of SCI, especially in terms of apoptosis and inflammatory response, and play a significant anti‐apoptotic and anti‐inflammatory role.^61^ This is consistent with the results of our study. Our study also found that MSC‐EVs could promote astrocyte conversion from A1 to A2, thereby exerting a role in promoting SCI repair.

It is well known that the microenvironment is different from that of normal cell culture in the injured state.^32^ After SCI, various cells in the tissue are in a state of ischemia and hypoxia, and the MSC‐EVs obtained under normoxic conditions in vitro cannot well simulate the injured microenvironment in vivo.^62^ Recently, it has been shown that H‐EV can effectively promote myocardial repair by transferring miR‐210.^9^ Therefore, we cultured MSCs under hypoxic conditions and then harvested their EV for the treatment of SCI rats. The results showed that H‐EV could play a significant role in the evaluation of motor function in behavior, as well as that of lesion size, apoptosis, and inflammatory response in histology and cytology. Importantly, we also simultaneously compared the efficacy of H‐EV with that of EV, and the results showed that the efficacy of H‐EV was better in terms of motor function repair, apoptosis, and inflammatory response after SCI compared with EV. In particular, we found that the H‐EV was also more effective in promoting the transformation of A1 astrocytes into A2 astrocytes than EV. Among them, on the 28th day after SCI, in the footprint analysis results, we found that compared with the EV group, the RI and BOS indexes in the PBS group and H‐EV group were not statistically significant efficacy. However, the H‐EV group had statistically significant efficacy in the RI and BOS compared to the PBS group. These results suggested that H‐EV could promote functional recovery of SCI in a later stage, while the efficacy of EV could not reach this extent. This result was also consistent with the results of the BBB score. When the BBB score was less than or equal to eight, SCI rats could not advance with the support of hind limbs, so hind limb footprints could not be collected in the Catwalk footprint analysis system, resulting in zero RI and BOS.^48^, ^49^ When the BBB score was greater than or equal to nine, SCI rats could move forward with the occasional or frequent support of hind limbs, so the Catwalk footprint analysis system could collect hind limbs footprints for RI and BOS analysis. In the results of RI and BOS in the PBS group, we found that the motor function of SCI rats could not recover to the extent of hind limb support forward. Compared with the PBS group, the motor function of SCI rats in the EV group showed no statistical significance in the indexes of RI and BOS, indicating that their motor function was difficult to recover to the extent of hind limb support. However, compared with the PBS group, the motor function of SCI rats in the H‐EV group showed significant statistical significance in RI and BOS indexes, indicating that their motor function could recover to the extent of hind limb support or even better. Based on our findings, we found that H‐EV was more effective than EV and could be a new therapy for SCI in the future.

Recent studies have shown that content miRNA plays an important role in the function of EV.^63^, ^64^ In the animal model of myocardial infarction, EV exerts anti‐apoptotic effects and promotes cardiac tissue and functional recovery by delivering miR‐21 to endothelial cells and cardiomyocytes.^65^ In the rat SCI model, EV loaded with miR‐29 transmits miR‐29 to nerve cell, promoting motor and sensory function repair.^66^ To further investigate the specific mechanism of the difference in efficacy between EV and H‐EV, we performed miRNA sequencing analysis of EV and H‐EV. It was found that the expression level of miR‐21 was significantly increased in H‐EV compared to EV. Thus, we incubated miR‐21‐overexpressed H‐EV and miR‐21‐knockdown H‐EV with reactive astrocytes, respectively, and the results showed that miR‐21 plays an irreplaceable role in H‐EV promoting the transformation of astrocytes from type A1 to type A2. It is concluded that H‐EV promotes SCI repair by delivering miR‐21 to convert A1 astrocytes into A2 astrocytes. This finding provides a strong rationale for the differential efficacy of EV and H‐EV, particularly in the role of altered astrocyte phenotypes. In order to further explore whether miR‐21 in H‐EV also plays a significant role in SCI rats, we administered miR‐21 up agomir, agomir NC, miR‐21 down antagomir, and antagomir NC via intravenous injection while simultaneously providing H‐EV treatment. The results of the BBB scoring and catwalk gait analysis suggested that miR‐21 can promote motor function recovery in SCI rats. In addition, the results of WB analysis confirm that miR‐21 indeed promotes the transition of astrocytes from the A1 phenotype to the A2 phenotype in SCI rats.

It has been shown that the JAK2/STAT3 signaling pathway is critical for astrocyte phenotypic changes. In the periventricular white matter (PWM) damage model of septic postnatal rats, melatonin could promote the conversion of A1 astrocytes into A2 astrocytes and ultimately played a neuroprotective role by up‐regulating the expression of JAK2/STAT3.^41^ Importantly, in the central nervous system (CNS), inhibiting the expression of astrocytes by ablation of STAT3 was detrimental to the repair of injury.^67^, ^68^ This result indicated that STAT3 also played an indispensable role in the process of injury repair. To better investigate the mechanism by which miR‐21 delivered by H‐EV affected phenotypic changes in astrocytes, we analyzed the expression levels of JAK2/STAT3 in the knockdown and overexpression miR‐21 groups by WB. It was found that the JAK2/STAT3 pathway was activated and expressed at increased levels in the miR‐21 overexpressing group. Meanwhile, the JAK2/STAT3 pathway was inhibited, and the expression level was decreased in the knockdown miR‐21 group. Therefore, we hypothesize that miR‐21 in H‐EV promotes astrocyte transformation from A1 type to A2 type by activating the JAK2/STAT3 signaling pathway. However, further inhibition of JAK2/STAT3 remains required to verify whether it is a target gene of miR‐21. In order to further validate the targeted regulation of JAK2/STAT3 by miR‐21, we conducted experiments using AG490, a specific inhibitor of JAK2/STAT3. The results revealed the ability of miR‐21 in H‐EV to enhance the expression levels of JAK2, P‐JAK2, STAT3, and P‐STAT3 was counteracted when AG490 was simultaneously administered. These findings provide clear evidence of the regulatory relationship between miR‐21 and JAK2/STAT3, demonstrating that miR‐21 can target the JAK2/STAT3 signaling pathway and regulate their expression levels. All in all, the efficacy differences observed in vivo between EV and H‐EV are not solely regulated by this single mechanism, the specific mechanism of action is complex, and the links between various miRNAs or proteins are ever‐changing. Our study only provides direction and ideas for future research, provides new options for the treatment of SCI, and complements the mechanism of action of EV.

## 5 CONCLUSIONS

In conclusion, our study suggests that both EV and H‐EV can promote SCI repair in vitro and in vivo. Both EV and H‐EV play an important role in the changes of astrocyte phenotype, inflammatory response, and apoptosis, and the effect of H‐EV is more prominent than that of EV. This result suggests that in clinical practice, H‐EV therapy given to patients with SCI can better reduce the inflammatory response, reduce apoptosis, and improve the prognosis of patients with SCI than EV therapy. Meanwhile, our study also found that H‐EV may regulate the JAK2/STAT3 pathway by delivering miR‐21 to promote the transformation of astrocytes from A1 to A2 phenotype, which provides a new target and idea for the treatment of SCI.

## AUTHOR CONTRIBUTIONS

Chunmei Chen and Chunhua Wang: Designed and guided the experiment and revised the manuscript. ZLY, ZYL, and JR: Completed most of the experiments and manuscript writing. HSX, MCZ, and XJX: Established SCI rat model. YKL and FBL: Analyzed data and typeset pictures. All the authors agreed on the final version of the manuscript.

## FUNDING INFORMATION

This work was sponsored by the Fujian Provincial Clinical Key Specialty Construction Program of Neurosurgery (Grant No. 05029002).

## CONFLICT OF INTEREST STATEMENT

The authors declare that they have no competing interests.

## Supporting information


Figure S1

## Data Availability

Most of the datasets supporting the conclusions of this article are included within this article and the additional files. The datasets used or analyzed during the current study are available upon reasonable request.
