# Supplementary material for: Hypoxic‐preconditioned mesenchymal stem cell‐derived small extracellular vesicles promote the recovery of spinal cord injury by affecting the phenotype of astrocytes through the miR‐21/JAK2/STAT3 pathway
Source: CNS Neurosci Ther. 2023 Aug 29;30(3):e14428. doi: 10.1111/cns.14428 (PMC10915983; doi:10.1111/cns.14428)
Supplement: Supplementary file 1 — Figure S1 [file CNS-30-e14428-s001.docx]

**
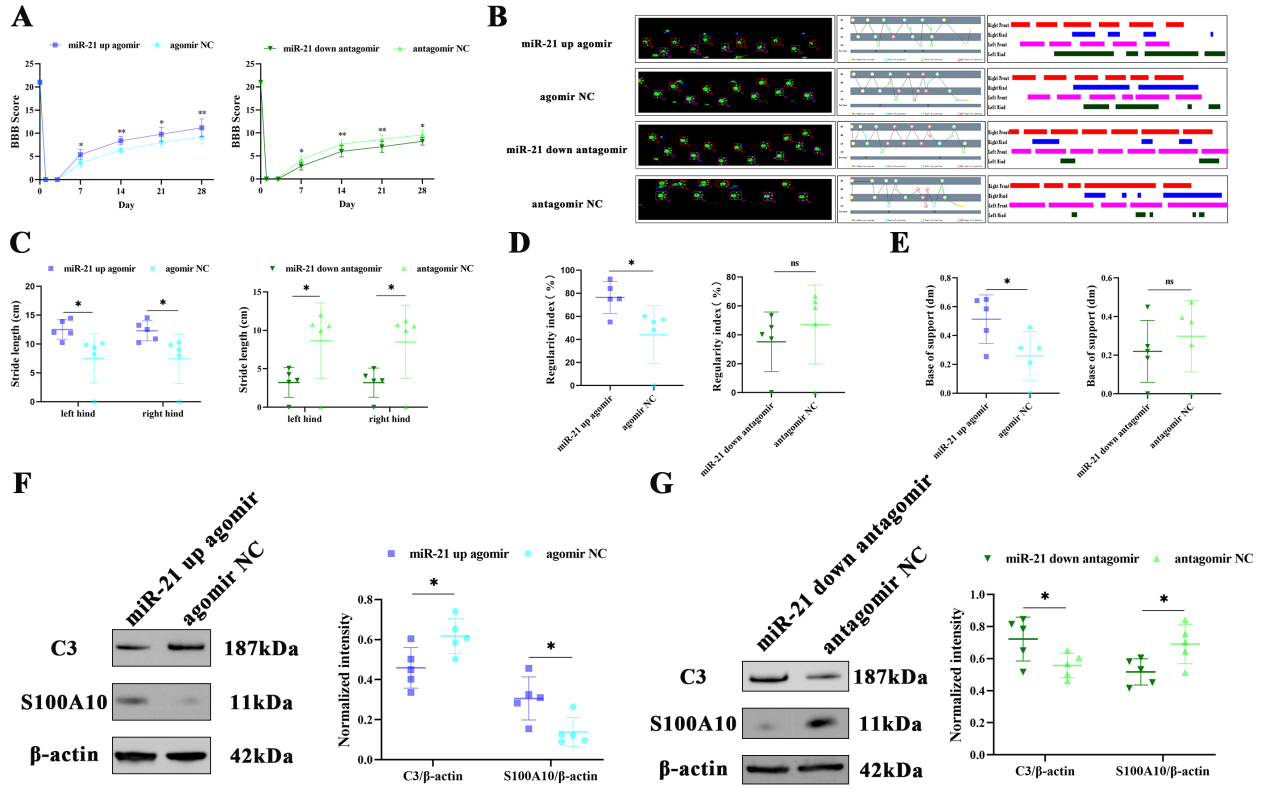
**

**Figure S1. miR-21 within H-EV enhances functional recovery and induces astrocyte phenotype switching from A1 to A2 after SCI *in vivo*.** (A) BBB score of miR-21 up agomir, agomir NC, miR-21 down antagomir, and antagomir NC groups on days 1, 3, 7, 14, 21, and 28 (n=5/group). (B) Representative Catwalk footprint analysis of miR-21 up agomir, agomir NC, miR-21 down antagomir, and antagomir NC groups at day 28 (n=5/group). (C-E) Three parameters of footprint analysis were used to quantify the motion function. (C) Stride length. (D) Regularity index. (E) The base of support. (F) Representative western blot analysis and relative quantitative of the expression levels of the spinal cord A1-related C3 and A2-related S100A10 protein markers in miR-21 up agomir and agomir NC groups (n=5/group, β-actin: internal reference protein). (G) Representative western blot analysis and relative quantitative of the expression levels of the spinal cord A1-related C3 and A2-related S100A10 protein markers in miR-21 down antagomir and antagomir NC groups (n=5/group, β-actin: internal reference protein). (*p<0.05, **p<0.01, ***p<0.001, ****p<0.0001, ns: non-significant)
